# Effects of sampling effort on biodiversity patterns estimated from environmental DNA metabarcoding surveys

**DOI:** 10.1038/s41598-018-27048-2

**Published:** 2018-06-11

**Authors:** Erin K. Grey, Louis Bernatchez, Phillip Cassey, Kristy Deiner, Marty Deveney, Kimberly L. Howland, Anaïs Lacoursière-Roussel, Sandric Chee Yew Leong, Yiyuan Li, Brett Olds, Michael E. Pfrender, Thomas A. A. Prowse, Mark A. Renshaw, David M. Lodge

**Affiliations:** 1 Division of Science, Mathematics and Technology, Governors State University, 1 University Parkway, University Park, Illinois 60484 USA; 2 Insitut de Biologie Intégrative et des Systèmes (IBIS), Université Laval, 1030 Avenue de la Médecine, Québec, G1V 0A6 Canada; 3 School of Biological Sciences, University of Adelaide, Adelaide, SA 5005 Australia; 4 Department of Ecology and Evolutionary Biology, Cornell University, 200 Rice Hall, Ithaca, NY 14853 USA; 5 South Australian Aquatic Sciences Centre, 2 Hamra Avenue, West Beach, SA 5024 Australia; 6 Fisheries and Oceans Canada, 501 University Crescent, Winnipeg, Manitoba R3T 2N6 Canada; 7 Tropical Marine Science Institute, National University of Singapore, 18 Kent Ridge Road, S2S Building, Singapore, 119227 Singapore; 8 Department of Biological Sciences, University of Notre Dame, 109b Galvin Life Science Center, Notre Dame, IN 46556 USA; 9 Oceanic Institute, Hawaii Pacific University, 41-202 Kalanianaole Highway, Waimanalo, HI 96795 USA; 10 Atkinson Center for a Sustainable Future, Cornell University, 200 Rice Hall, Ithaca, NY 14853 USA; 11 Environmental Change Initiative, University of Notre Dame, 1400 East Angela Boulevard, Unit 117, South Bend, IN 46617 USA; 12 School of Mathematical Sciences, University of Adelaide, Adelaide, SA 5005 Australia

**Keywords:** Biodiversity, Molecular ecology

## Abstract

Environmental DNA (eDNA) metabarcoding can greatly enhance our understanding of global biodiversity and our ability to detect rare or cryptic species. However, sampling effort must be considered when interpreting results from these surveys. We explored how sampling effort influenced biodiversity patterns and nonindigenous species (NIS) detection in an eDNA metabarcoding survey of four commercial ports. Overall, we captured sequences from 18 metazoan phyla with minimal differences in taxonomic coverage between 18 S and COI primer sets. While community dissimilarity patterns were consistent across primers and sampling effort, richness patterns were not, suggesting that richness estimates are extremely sensitive to primer choice and sampling effort. The survey detected 64 potential NIS, with COI identifying more known NIS from port checklists but 18 S identifying more operational taxonomic units shared between three or more ports that represent un-recorded potential NIS. Overall, we conclude that eDNA metabarcoding surveys can reveal global similarity patterns among ports across a broad array of taxa and can also detect potential NIS in these key habitats. However, richness estimates and species assignments require caution. Based on results of this study, we make several recommendations for port eDNA sampling design and suggest several areas for future research.

## Introduction

Global biodiversity surveys are crucial for understanding the impacts of changes in climate and human activity but can be logistically difficult to standardize across many taxa and sites. Port ecosystems are hotspots of harmful aquatic invasions^1^ and subject to changes in coastal land use and global shipping patterns influenced by trade policies, infrastructure development, and climate-driven changes in sea ice, salinity, and temperature. Currently our knowledge of patterns and processes driving invasions in these ecosystems is limited due to challenges associated with traditional survey methods (e.g., SCUBA, settlement plates, plankton tows, and benthic trawls), including difficulties in port access and low capture rates for cryptic or rare species. Thus, few comprehensive port surveys exist, and those that do are mainly limited to larger organisms^1^.

Environmental DNA (eDNA) metabarcoding surveys have proven useful for many ecosystems and could potentially overcome the limitations of traditional port surveys. Aquatic eDNA can be shed from feces, scales, gametes, or other extra-organismal sources of DNA suspended in water^2^. Sampling eDNA requires collection of water in the field, which can be used to metabarcode a broad suite of species using general primers and high-throughput sequencing. Recent studies in coastal marine ecosystems have demonstrated the efficacy of this method to describe biodiversity^3^. For example, Thomsen *et al*.^4^ and Yamamoto *et al*.^5^ detected higher fish richness with eDNA metabarcoding compared to traditional methods, Kelly *et al*.^6^ demonstrated a link between eel-grass metazoans and coastal urbanization with eDNA metabarcoding, Ardura *et al*.^7^ used eDNA metabarcoding to track species transport in ballast water, and Borell *et al*.^8^ used eDNA metabarcoding to identify three invasive invertebrates in Bay of Biscay ports. Clearly, eDNA metabarcoding shows great promise for understanding biodiversity and detecting species transported by shipping.

Standardized port eDNA metabarcoding surveys could greatly increase our understanding of biodiversity in these dynamic, globally-connected habitats. However, developing a standardized protocol that is applicable globally is challenging because ports vary considerably in size, complexity, hydrodynamics, physical structures, and benthic substrates. Variation in eDNA sampling collection, extraction, and sequencing methods can complicate comparison of samples from different projects^9^. Even when sampling methods are identical, an increase in sampling effort almost inevitably yields more species collected^10^. Sampling effort variation can therefore confound comparisons of species richness and community similarity even among studies using similar methods^11^ and, if not adequately considered, prevent accurate understanding of global biodiversity.

This study’s goal was to apply an eDNA metabarcoding survey method for metazoans (multicellular animals) to ports and determine how primer set and sampling effort influences global biodiversity patterns and nonindigenous species (NIS) detection. We sampled eDNA from surface waters in four ports with inexpensive and quick collection methods, and used two universal metazoan primer sets, 18 S and COI, to make the survey taxonomically broad. To optimize sampling effort for future port surveys, we explored how eDNA collection effort and sequencing depth influenced biodiversity metrics. Lastly, we evaluated the ability of each primer set to detect both known and un-recorded potential NIS. Our results support multiple recommendations for standardizing eDNA metabarcoding sampling effort in future port eDNA metabarcoding surveys.

## Results and Discussion

**Figure 1:**
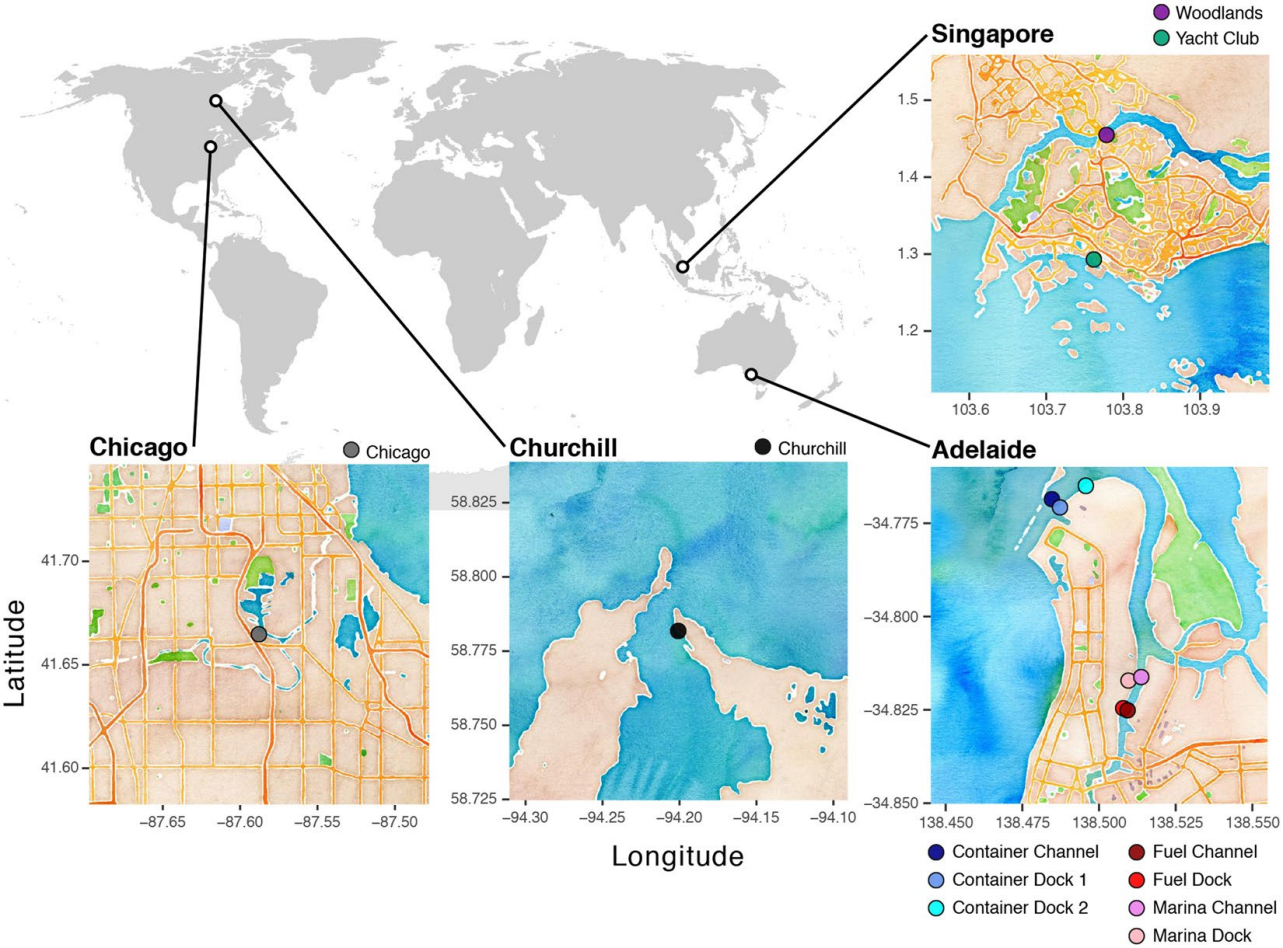
Map of sites sampled for this study. Maps were generated with the ggmap package version 2.6.1^36^ in R programming language version 3.2.2^37^ using map tiles by © Stamen Design, under CC BY 3.0. (https://creativecommons.org/licenses/by/3.0/), with data by OpenStreetMap, under CC BY SA (https://creativecommons.org/licenses/by-sa/3.0/). This figure is not covered by the CC BY license.

A total of 146 eDNA samples were collected across four ports. The number of samples per site and the number of sites differed among ports (Fig. 1) for logistical reasons. At Chicago, USA, 20 samples were taken at one site on 20 November 2013 from a dock. In Churchill, Canada, 20 samples were taken at one site on 13 August 2015 from a beach near a dock at slack high tide. In Singapore, 40 samples were taken at two sites on 11 July 2014 from docks (n = 20 per site) during flood tide. In Adelaide, Australia, 66 samples were taken at low tide at 7 sites on 3 July 2014 from a boat, with four sites sampled within two meters of a dock and three sites sampled in the middle of the channel (n = 9 or 10 per site). Churchill eDNA samples had slightly different collection, DNA extraction, and sequencing protocols than those from other ports, but all sample sequences were trimmed, clustered, and assigned to taxa using the same bioinformatics pathway (see Supplementary Methods).

### Taxonomic Coverage of Primer Sets

Clustering and filtering yielded 8,525 18 S and 11,872 COI molecular operational taxonomic units (MOTUs) across all samples, of which 13% of 18 S (1,117) and 39% of COI (4,605) MOTUs were assigned to a metazoan phylum (metMOTUs; see Supplementary Table S1 for sample and site sequencing summaries). No-template control filtering removed reads from 52 18 S and 245 COI metMOTUs from all field samples. Cooler blank filtering removed three reads from two COI metMOTUs from Chicago samples, six reads from one 18 S and 15 reads from 5 COI metMOTUS from Singapore samples, and 43 reads from three 18 S and 30,846 reads from 18 COI metMOTUs from Adelaide samples (the latter read count being dominated by common dust mite *Dermatophagoides pteronyssinus*). All 11 mock species were sequenced and correctly assigned in the COI data, while only three mock species were sequenced and none correctly assigned in the 18 S data.

**Figure 2:**
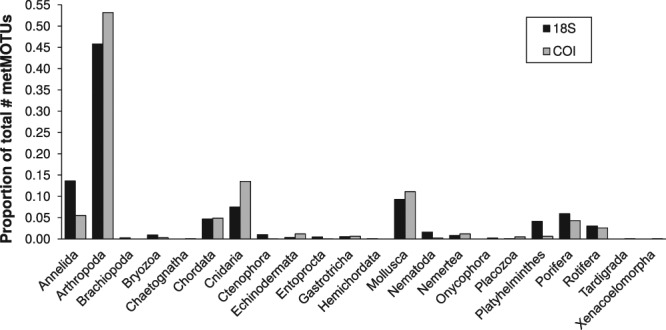
Proportion of metazoan MOTUs in each phylum for the 18 S (black) and COI (grey) datasets.

COI primers produced more metMOTUs, but many of these had weak taxonomic assignments. Pooling metMOTUs across all ports and using only those with assignments with > 90% sequence coverage and identity yielded 795 18 S metMOTUs spanning 18 phyla and 600 COI metMOTUs spanning 11 phyla (Fig. 2). While COI lacked 7 minor phyla (Brachiopoda, Ctenophora, Entoprocta, Hemichordata, Nematoda, Nemertea, and Placozoa) and had relatively more Chordate metMOTUs than 18 S, both primer datasets were dominated by Arthropoda metMOTUs and had similar proportions for Annelida, Cnidaria, Mollusca, Porifera, and Rotifera.

Overall, the COI primers successfully retrieved all mock fish species and yielded more metMOTUs than 18 S, which is similar to the findings of Borrell *et al*.^8^. However, many of the COI metMOTUs had low quality taxonomic assignments. After filtering metMOTUs based on assignment quality, the taxonomic coverage for the 18 S primer set was higher than that of COI, indicating a trade-off between metMOTU abundance and assignment quality in these primers. Despite the differences, relative metMOTU abundances in major metazoan phyla were similar with both primer sets.

### Variation in Sequencing and eDNA Collection Effort

Sequencing effort differed among samples, but general patterns were apparent (see Supplementary Figures S1 and S2 and Tables S2 and S3 for with-sample rarefaction curves and richness estimates). Churchill, Chicago, and Singapore Woodlands were sequenced at the shallowest depth for both primers (average <50,000 reads per sample), while other sites averaged ~75,000–190,000 reads per sample. Within-sample rarefaction curves did not plateau in Churchill samples (<20,000 reads per sample) but began to plateau at ~25,000 reads in Chicago and Singapore Woodlands samples and ~100,000–150,000 reads in samples from all other sites. An average of 80.8% and 78.6% of Chao1 estimated metMOTUs were recovered per 18 S and COI sample, respectively, with Churchill samples having the lowest completeness (74% and 73% of Chao1 estimate for 18 S and COI, respectively).

**Figure 3:**
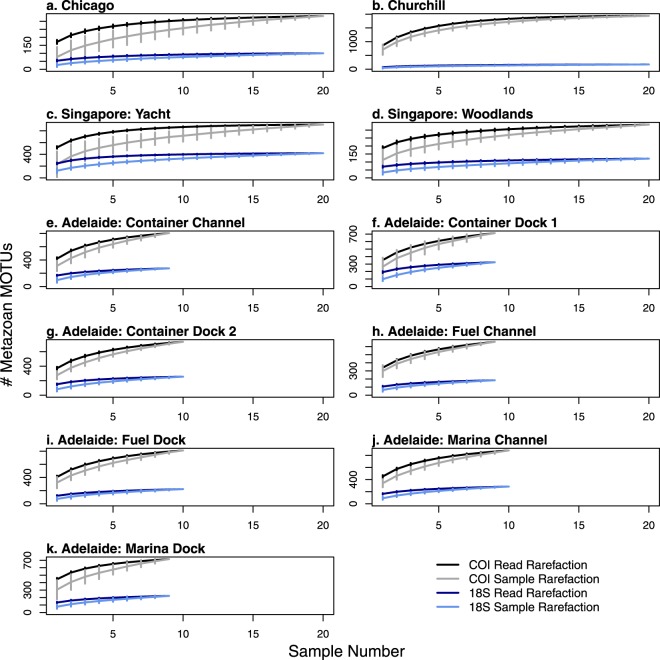
Rarified metMOTU accumulation curves by reads and samples for each site. Solid black line denotes COI read rarefaction, grey line denotes COI sample rarefaction, dark blue line denotes 18 S read rarefaction, and light blue line denotes 18 S sample rarefaction. Read curves were plotted on the x-axis using the average number of reads per sample. Errors bars represent 95% confidence intervals.

Variation in eDNA collection effort existed among sites as well. 18 S sample rarefaction curves plateaued at Chicago, Churchill and both Singapore sites at 5–15 samples while COI curves plateaued at 15–20 samples at these sites (Fig. 3). Adelaide curves, which had only 9 or 10 samples each, did not plateau in either 18 S or COI curves. Aggregation of metMOTUs within samples, as indicated by sample curves falling below read curves, was apparent in Singapore Yacht 18 S, Singapore Yacht COI, Singapore Woodlands COI, and Chicago COI curves (Fig. 3). This pattern, typically attributed to spatial aggregation of species in traditional surveys, could here be due to either spatial aggregation of metazoan eDNA in port surface waters or variation in PCR reactions among samples. Further experimentation is needed to tease apart these non-exclusive factors.

### Biodiversity Patterns

**Figure 4:**
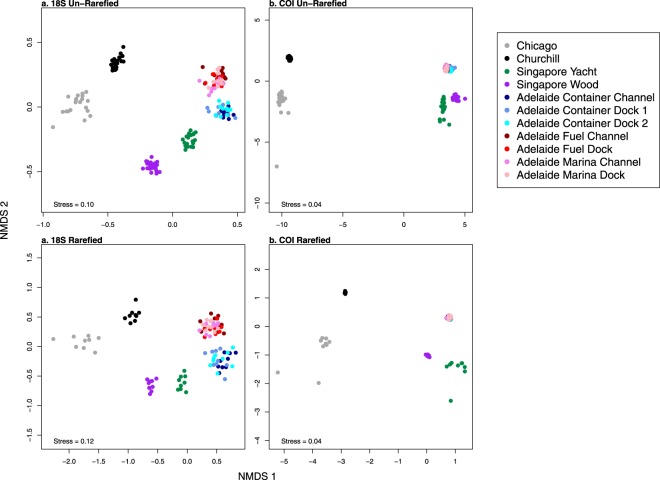
Ordination of (**a**) 18 S un-rarefied (**b**) COI un-rarefied, (**c**) 18 S rarefied, and (**d**) COI rarefied datasets and using non-metric multidimensional scaling of Chao dissimilarity estimates. Samples are colored by site and ordination stress values are given on each plot.

**Figure 5:**
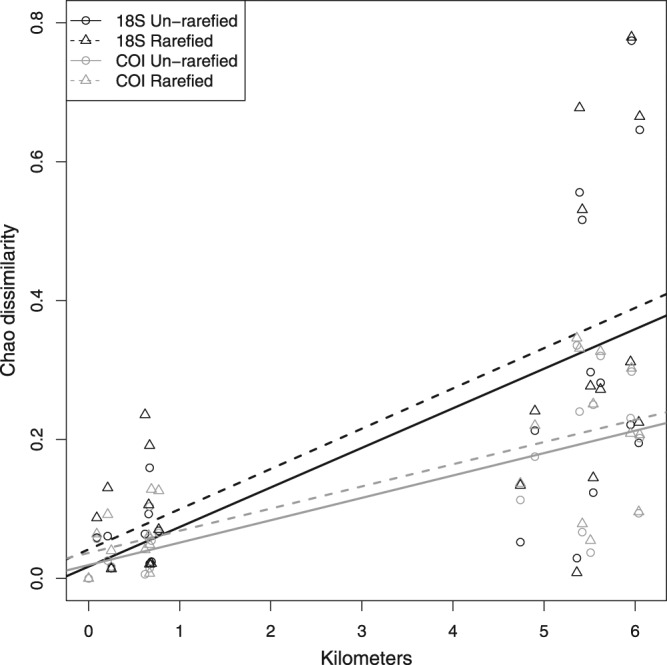
Between-site Chao dissimilarity by over-water distance for seven Adelaide sites. Linear regression lines for each primer-rarefaction combination are shown. Mantel tests were significant at the *p* ≤ 0.02 level for each of the four dissimilarity by distance correlations (see text).

Dissimilarity ordinations consistently showed that samples grouped by port, with samples from each port forming a unique cluster in all datasets (Fig. 4). Adelaide and Singapore clusters were closer to each other than to Chicago, and the Churchill cluster, which followed different protocols, was closest to Chicago in all ordinations. Within sites, 18 S dissimilarities were larger than COI dissimilarities, but the overall pattern between sites was consistent. Samples from the two Singapore sites, located on opposite sides of the island, were distinct from each other with no overlap in any ordination. Adelaide seaward sites (Container Channel, Container Dock 1, Container Dock 2) formed a cluster unique from the four upriver sites (Fuel Channel, Fuel Dock, Marina Channel, Marina Dock) in the 18 S but not the COI ordination. Samples from sites within the two Adelaide clusters were intermixed with each other, suggesting that eDNA is well-dispersed at the scale of about 500 m-1 km. Also consistent across datasets was a significant positive correlation between Adelaide site dissimilarities and geographic distance (Fig. 5; 18 S un-rarefied r = 0.56, *p* = 0.02; COI un-rarefied r = 0.77, *p* < 0.01; 18 S rarefied r = 0.58, *p* = 0.02; COI rarefied r = 0.74, *p* < 0.01), which was expected given the estuarine gradient of this river port.

**Figure 6:**
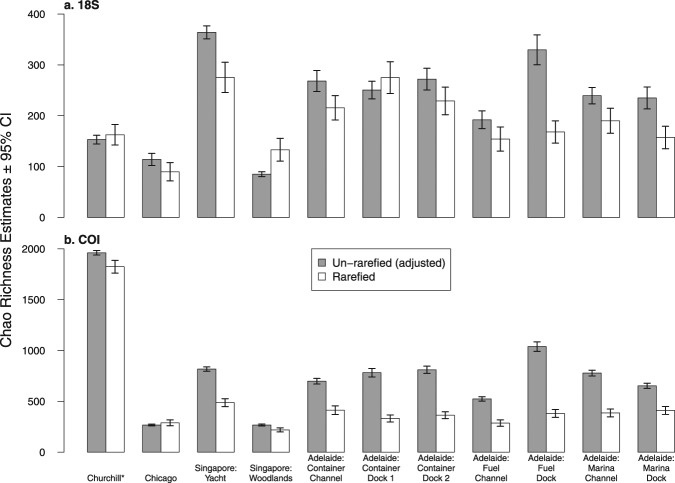
Site metMOTU Chao2 richness estimates at 20 samples from the (**a**) 18 S dataset and (**b**) COI dataset. Grey bars represent estimates from the un-rarefied, singleton-adjusted dataset and white bars from the rarefied dataset. Error bars represent 95% confidence intervals. *Churchill samples were collected and sequenced using a different method and so cannot be compared to the other sites.

Unlike community similarity patterns, site metMOTU richness estimates were inconsistent across barcodes and sampling effort (Fig. 6). Un-rarefied richness estimates were generally higher than those from rarefied data, except in three cases (Singapore Woodlands 18 S, Adelaide Container Dock1 18 S, and Chicago COI). Of 11 sites, un-rarefied and rarefied 95% confidence intervals overlapped at only four sites in the 18 S dataset and one site in the COI dataset, with notable differences in the COI estimates at the Singapore Yacht and all Adelaide sites. Richness rankings among the non-Churchill sites varied between barcodes and methods, but ranking correlations were significant in all cases (18 S un-rarefied and rarefied Spearman ρ = 0.83, p = 0.001; COI un-rarefied and rarefied ρ = 0.55, p = 0.05; un-rarefied 18 S and COI ρ = 0.94, p =  < 0.001; rarefied 18 S and COI ρ = 0.62, p = 0.03). Churchill COI richness estimates were much higher than the other sites, perhaps due to differences in eDNA collection (e.g., the use of glass-fiber filter membranes in Churchill versus cellulose nitrate membranes in other ports), extraction (e.g. use of phenol chloroform for Churchill versus chloroform for other samples), or amplification protocols (e.g. use of a single annealing temperature for Churchill COI amplifications versus a touchdown program for other amplifications) at this site (Supplementary Methods).

Overall, we found that community dissimilarity patterns and dissimilarity-distance correlations were robust to barcode and sampling effort (Figs 4, 5), while site metMOTU Chao2 richness estimates were not (Fig. 6). The latter finding is consistent with Haegeman *et al*.^12^. who found that reliable bacterial MOTU richness estimates are challenging due to spurious singletons, unknown underlying MOTU abundance distributions, and the reliance of non-parametric estimators on singleton frequencies. Although we attempted to correct for spurious singletons, site metMOTU richness estimates were still variable among our datasets, indicating that they are not robust to the sequencing and collection effort variation in this study.

### NIS Detections

This survey detected several known and un-recorded potential NIS, but some assignment similarity metrics were weak, particularly in the COI dataset (see Supplementary Tables S4 and S5). In Chicago, seven known NIS were detected: two with 18 S (quagga mussel *Dreissena rostriformis* and copepod *Eurytemora affinis*) and five with COI (white perch *Morone Americana*, common carp *Cyprinus carpio*, Asian clam *Corbicula fluminea*, copepod *Eurytemora carolleeae*, and European earthworm *Lumbricus rubellus*), all with high sequence similarity (coverage = 100%, identity >97%) except *D. rostriformis* (coverage = 100%, identity = 89%). In Adelaide, 11 known NIS or cryptogenic species were detected: five with both primer sets (ascidian *Styela plicata*, green crab *Carcinus maenus*, hydrozoans *Plumularia setacea* and *Coryne eximia*, Senhouse mussel *Musculista senhousia*, and polychaete *Hydroides “elegans”*), one with only 18 S (ascidian *Ciona inestinalis*), and four with only COI (bryozoans *Tricellaria occidentalis* and *Watersipora arcuata*, Chameleon Goby *Tridentiger trigonocephalus*, Mediterranean mussel *Mytilus galloprovincialis*). All Adelaide 18 S and six COI NIS assignments were strong (>99% coverage and identity) while four COI assignments were weak ( < 95% coverage or identity).

Un-recorded potential NIS included eight 18 S metMOTUs found in all ports, and 25 18 S and 13 COI metMOTUs found in three ports. All-port 18 S metMOTUs consisted mostly of plausible NIS, including five rotifers (two of which, *Synchaeta pectinata* and *Cephalodella forficula* are cosmopolitan), a cosmopolitan hydroid (*Bougainvilla muscus*), a cosmopolitan flatworm (*Microstomum lineare*), and human. The 25 three-port 18 S metMOTUs spanned 9 phyla and many had cosmopolitan distributions. Except for one sponge assignment with low similarity (*Spongionella cf. foliascens*) all of the three-port 18 S assignments had coverages >98.5% and identities > 95%. In the COI dataset, the 13 three-port metMOTUs spanned five phyla and generally had weak assignments (coverage <90% or identity <90%), with three exceptions: the feral pig *Sus scrofa* (coverage and identity = 100%), cladoceran *Macrothrix sp*. HE-364 (coverage = 100%, identity = 99%), and sponge *Haliclona aculata* (coverage = 100%, identity = 99%). All three of these taxa have cosmopolitan distributions; however, *S. scrofa* is also a common laboratory contaminant^13^.

Overall, many plausible NIS were identified by comparing assignments to port NIS checklists or by investigating assignments found in three or more ports. More recorded NIS were detected with COI (14) than with 18 S (8), but more metMOTUs shared between three or more ports, which represent potential but currently un-recorded NIS, were found with 18 S (33 with 18 S versus 13 with COI). However, several assignments were likely erroneous with low sequence coverages or identities, particularly in the COI dataset. Further, 18 S is well known to be more conserved among many metazoan clades^14^, indicating that metMOTUs shared between three or more ports may truly be different species. Further testing of universal metazoan barcodes against well-curated sequence databases and port species checklists is sorely needed to better determine the benefits and drawbacks of each barcode.

### Summary and Recommendations

In summary, we detected eDNA from at least 18 metazoan phyla in ports and our analyses give us confidence that the methods used here can reliably estimate community dissimilarity patterns and identify plausible NIS without the extensive fieldwork and taxonomic expertise required by traditional surveys. Although richness estimates and some taxonomic assignments are unreliable, we conclude that eDNA metabarcoding can potentially transform our understanding of port biodiversity in the face of global change. For example, applying this survey to more ports over time could reveal changes in port species composition dissimilarities, allowing us to tease apart the effects of climate and shipping on biodiversity in these key hotspots of invasion and other anthropogenic change.

Based on our results, we make the following recommendations for future port eDNA metabarcoding surveys and research:

1. Protocols: Standardize eDNA collection, extraction, and sequencing protocols to maximize biodiversity pattern inference across sites. Here we used two sets of protocols, one for Churchill and one for the other three ports (see Supplementary Methods), which prevented direct comparison of biodiversity metrics between Churchill and the other ports. Further research and conversation among practitioners is needed to determine the optimal set of protocols for port eDNA surveys. We suggest that both sets of protocols used here provide a good starting point for these efforts.
2. Primer Choice: For biodiversity estimation, both the COI and 18 S primer sets yielded similar taxonomic breadth and dissimilarity patterns (Figs 2, 4 and 5), so either or both could be effective for this aim. To optimize NIS detection, we recommend using multiple primers, as the two primers in this survey detected different NIS (Supplementary Dataset S3). For eDNA surveys targeted at specific NIS that are known beforehand (which was not the case in this study), species-specific quantitative or digital droplet PCR assays will be more sensitive than metabarcoding^15^.
3. Sequencing Depth: Sequencing depth recommendations vary depending on the purpose of the survey. For community dissimilarity estimation, read depths of 10,000 and 40,000 reads per sample are needed for the 18 S and COI primers used in this study, respectively. For species richness estimates or NIS detection, sequencing each sample at a depth of 150,000 reads will yield ~80% of estimated richness in most samples for both primer sets (Supplementary Dataset S2). The depth needed for less diverse sites or more specific primers is probably lower, but this should be evaluated beforehand by over-sequencing a few samples.
4. eDNA Sample Collection Effort: Given the observed heterogeneity of metMOTUs across samples within some sites (Fig. 3), we recommend collecting at least 9 × 250 mL samples per site to estimate community dissimilarity and 15 samplers for metMOTU richness estimation, with samples taken about every 2–4 meters in a site. Further research is needed to determine how much of this heterogeneity is due to PCR variation versus spatial aggregation of eDNA.
5. Number of Sites within a Port: Multiple sites will need to be sampled to capture the full biodiversity of a port (Fig. 4). Based on a dissimilarity by distance analysis for seven Adelaide sites (Fig. 5), we recommend that sites be located about 0.5–1 km apart.
6. Species Assignment Accuracy: Species assignments can be informative but should be treated with caution (see Supplementary Dataset S3) given known errors and omissions in sequencing and reference libraries. Any potential NIS detected with eDNA metabarcoding should therefore be confirmed with traditional surveys or species-specific qPCR or ddPCR surveys. Additional species lists for ports (and many other coastal habitats) and more complete and accurate sequence databases would enable better evaluation of eDNA metabarcoding survey accuracies.

## Methods

### eDNA Collection, Extraction and Amplicon Sequencing

eDNA collection, extraction and amplicon sequencing protocols differed between Churchill and the other ports (Supplementary Methods). For all ports, a sample consisted of 250 mL of surface water. Samples from Chicago, Adelaide, and Singapore were stored on ice immediately after collection and eDNA was captured in the lab by filtering through cellulose nitrate membranes (47 mm diameter, 0.45 µm pore-size) within 8 hours of collection. Churchill samples were filtered immediately in the field with a syringe and glass-fiber membranes (25 mm diameter, 0.7 µm pore-size). After filtration, all membranes were stored in a sterile microtube with 700 µl of Longmire’s buffer^16^.

DNA was extracted from the Chicago, Singapore, and Adelaide samples using a chloroform protocol. Amplicon sequencing included an initial 50 μL PCR using primers with 5′ tail sequences corresponding to part of the Nextera® adaptors and a second PCR to attach library specific indices and remaining Nextera® sequences. DNA was extracted from the Churchill samples using a QIAshredder (Qiagen) and phenol-chloroform protocol. Churchill amplicon sequencing involved one PCR with three 24 μL replicates per sample using barcode primers tailed on the 5′ end with the entire Nextera® adaptors.

Both protocols amplified the same two barcode sequences [a 313 bp COI fragment using the MlCOIintF^17^ and jgHCO2198^18^ primers and a ribosomal 18 S gene fragment (~ 378 bp) using the 18S_574F and 18S_952R primers^19^] and sequenced on an Illumina MiSeq platform (Illumina, San Diego) using a paired-end MiSeq Reagent Kit V3 (sequence length = 300 bp) following manufacturer’s instructions.

### Bioinformatics and Contamination Controls

Raw sequence reads were filtered based on their quality, merged, and clustered into molecular operational taxonomic units (MOTUs) at 97% similarity^20^ (Supplementary Methods). MOTUs were assigned to taxa in the NCBI NR database with two different approaches: SAP v1.9.3^21^ and the BLAST function in Geneious v9.1.5^22^. For all analyses we used only MOTUs that were assigned to the metazoan phylum (metMOTUs) by either assignment method, using the SAP assignment when the two methods disagreed.

Following recommended eDNA control protocols^23^, we used cooler blanks as field controls; for laboratory controls, we used mock communities and no-template controls at each step of extraction and PCR (Supplementary Methods). To remove contaminate MOTUs from the data, we subtracted contaminant reads from field samples^24^ as follows: mock MOTU read counts were subtracted from each field sample in the same sequencing run, cooler blank MOTU read counts were subtracted from each field sample transported in the same cooler, and no-template MOTU read counts were subtracted from all field samples.

### Variation in eDNA Collection and Sampling Effort

Differences in eDNA sampling effort can occur at several stages^25^. We explored two types of effort that could differ among samples taken with the same protocol: sequencing effort, which is the number of reads generated per sample, and eDNA collection effort, which depends on the volume of water collected, metMOTU diversity, and spatial distribution of eDNA in the site^26^. To investigate variation in sequencing effort, we generated read rarefaction curves for each sample to determine if and when curves plateaued; the latter indicating all metMOTUs in the sample were sequenced. To estimate the sequencing completeness of each sample, we divided the number of observed metMOTUs by the Chao1 richness estimate^27^ for metMOTUs for that sample. We explored variation in eDNA collection effort among sites by plotting site-specific rarefied sample curves for each site to observe if and when curve plateaued. To investigate spatial aggregation of metMOTUs within a site, we plotted rarefied pooled read curves for each site along with the rarefied sample curves. Sample curves will increase more slowly than read curves when metMOTUs are aggregated within samples, with greater aggregation yielding a relatively slower increase in sample curves^28^. Sample rarefaction curves, sample Chao1 estimates, site sample rarefaction curves, and site read rarefaction curves were calculated with the R package *vegan*^29^, using the *rarecurve*, *estimateR, specaccum (*method = “random”), and specaccum (method = ”rarefaction”) functions respectively.

### Biodiversity Metrics

When sampling effort differs among samples or sites, two common approaches for comparing biodiversity metrics exist: 1) rarefy the data to the lowest effort, or 2) use non-parametric estimates that are robust to unequal sampling efforts. The rarefaction approach is compatible with many biodiversity metrics but often requires omission of a substantial amount of data. Non-parametric estimators are more robust to effort variation, but they can be biased at low effort levels and yield wide confidence intervals^11^. To explore how sequencing and collection effort influenced biodiversity patterns in this survey, we compared non-parametric community dissimilarity and richness estimates from un-rarefied data (where sequencing and collection effort varied among samples and sites) to those from rarefied data (where all samples had the same number of reads and all sites had the same number of samples). We rarefied by selecting 9 samples (the lowest sample number per site) with the highest read counts for each site, and then randomly selected reads without replacement from each sample up to the lowest observed read count (lowest read count 18 S = 9,081, COI = 40,401). This comparison allowed us to infer the effect of sequencing and collection effort on non-parametric biodiversity metrics and to determine if these metrics are robust across barcodes and effort levels.

We then compared three biodiversity patterns across primer and un-rarefied/rarified datasets: between-sample community dissimilarity, correlation between site dissimilarity and geographic distance, and site metMOTU richness. We estimated between-sample dissimilarities using the Chao dissimilarity index, which is similar to the Jaccard index except that it accounts for unseen metMOTUs shared between samples^30^, and visualized these dissimilarities with non-metric multidimensional ordination (NMDS). We evaluated the correlation between site Chao dissimilarities and over-water distance in Adelaide, a river port with 7 sites distributed along several kilometers (Fig. 1), with a Mantel test and visualized the correlation by plotting site dissimilarity by distance and adding linear regression lines for each primer set-rarefaction combination. Chao dissimilarities and NMDS ordinations were calculated using the *vegan* functions *metaMDS* and *ordiplot*, respectively. To calculate Adelaide site Chao dissimilarities we pooled reads from all samples in a site and used the *vegan* function *vegdist*.

To estimate metMOTU richness, we first adjusted the number of singletons (number of metMOTUs with one read per site) in each un-rarefied sample to correct for spurious sequences using the algorithm provided in Chiu and Chao^31^. We estimated metMOTU richness at 20 samples using the *estimateD* function in the R package *iNEXT*^32,33^, setting Hill number *q* = 0. A one-tailed Spearman rank correlation tested for concordance between site richness rankings between the different barcodes and between un-rarefied and rarefied datasets.

Because Churchill samples were filtered, extracted, and amplified differently than those from the other ports (Supplementary Methods), we did not compare its metMOTU richness with that of other ports. However, we did compare relative dissimilarity between Churchill samples and other ports.

### Nonindigenous Species (NIS) Detection

In addition to revealing global biodiversity patterns, eDNA metabarcoding may also detect NIS in ports. However, errors and omissions in reference databases^34^ or sequences require caution for any species assignment. To assess this survey’s ability to identify NIS, we checked species assignments from Chicago and Adelaide against NIS species lists for these ports (Chicago: Great Lakes Aquatic Nonindigenous Species Information System www.glerl.noaa.gov/res/Programs/glansis; Adelaide: Wiltshire *et al*.^35^). Next, we evaluated our ability to detect unrecorded NIS by evaluating metMOTUs found in three or more ports, as species were unlikely to have dispersed naturally to at least one of any three ports in this study. For both analyses, we assessed whether an assignment was a true NIS based on percent of the metMOTU sequence that overlapped with the assignment sequence (% coverage), the extent to which the metMOTU and the assignment have the same nucleotides at the same positions (% identity), and the known global distribution of the species derived from the World Register of Marine Species (www.marinespecies.org) or the IUCN Red List (www.iucnredlist.org).

## Electronic supplementary material

Supplementary Methods

Supplementary Information

## Data Availability

Raw sequences for all samples have been deposited in NCBI’s Sequence Read Archive (SRA, http://www.ncbi.nlm.nih.gov/), with Chicago, Singapore and Adelaide sequences under BioProject PRJNA3955904 and Churchill sequences under BioProject PRJNA388333. Filtered MOTU data and R scripts for biodiversity analyses are freely available on Dryad at 10.5061/dryad.40782nd.

## References

[1] Ruiz GM, Carlton JT, Grosholz ED, Hines AH (1997). Global Invasions of Marine and Estuarine Habitats by Non-Indigenous Species: Mechanisms, Extent, and Consequences. Integr Comp Biol.

[2] Creer S (2016). The ecologist’s field guide to sequence-based identification of biodiversity. Methods Ecol Evol.

[3] Deiner Kristy, Bik Holly M., Mächler Elvira, Seymour Mathew, Lacoursière‐Roussel Anaïs, Altermatt Florian, Creer Simon, Bista Iliana, Lodge David M., Vere Natasha, Pfrender Michael E., Bernatchez Louis (2017). Environmental DNA metabarcoding: Transforming how we survey animal and plant communities. Molecular Ecology.

[4] Thomsen PF (2012). Detection of a Diverse Marine Fish Fauna Using Environmental DNA from Seawater Samples. PLOS ONE.

[5] Yamamoto S (2017). Environmental DNA metabarcoding reveals local fish communities in a species-rich coastal sea. Scientific Reports.

[6] Kelly RP (2016). Genetic signatures of ecological diversity along an urbanization gradient. PeerJ.

[7] Ardura A (2015). Environmental DNA evidence of transfer of North Sea molluscs across tropical waters through ballast water. J Molluscan Stud.

[8] Borrell YJ, Miralles L, Huu HD, Mohammed-Geba K, Garcia-Vazquez E (2017). DNA in a bottle—Rapid metabarcoding survey for early alerts of invasive species in ports. PLOS ONE.

[9] Deiner K, Walser J-C, Mächler E, Altermatt F (2015). Choice of capture and extraction methods affect detection of freshwater biodiversity from environmental DNA. Biological Conservation.

[10] Evans Nathan T., Li Yiyuan, Renshaw Mark A., Olds Brett P., Deiner Kristy, Turner Cameron R., Jerde Christopher L., Lodge David M., Lamberti Gary A., Pfrender Michael E. (2017). Fish community assessment with eDNA metabarcoding: effects of sampling design and bioinformatic filtering. Canadian Journal of Fisheries and Aquatic Sciences.

[11] Gotelli NJ, Colwell RK (2001). Quantifying biodiversity: procedures and pitfalls in the measurement and comparison of species richness. Ecology Letters.

[12] Haegeman B (2013). Robust estimation of microbial diversity in theory and in practice. ISME J.

[13] Leonard JA (2007). Animal DNA in PCR reagents plagues ancient DNA research. Journal of Archaeological Science.

[14] Tang CQ (2012). The widely used small subunit 18S rDNA molecule greatly underestimates true diversity in biodiversity surveys of the meiofauna. Proceedings of the National Academy of Sciences.

[15] Simmons M (2015). Active and passive environmental DNA surveillance of aquatic invasive species. Canadian Journal of Fisheries and Aquatic Sciences.

[16] Longmire JL, Maltbie M, Baker RJ (1997). Use of lysis buffer in DNA isolation and its implications for museum collections. Occasional Papers of the Museum of Texas Tech University.

[17] Leray M (2013). A new versatile primer set targeting a short fragment of the mitochondrial COI region for metabarcoding metazoan diversity: application for characterizing coral reef fish gut contents. Frontiers in Zoology.

[18] Geller J, Meyer C, Parker M, Hawk H (2013). Redesign of PCR primers for mitochondrial cytochrome c oxidase subunit I for marine invertebrates and application in all-taxa biotic surveys. Mol Ecol Resour.

[19] Hadziavdic K (2014). Characterization of the 18S rRNA Gene for Designing Universal Eukaryote Specific Primers. PLOS ONE.

[20] Edgar RC (2013). UPARSE: highly accurate OTU sequences from microbial amplicon reads. Nat Meth.

[21] Munch K, Boomsma W, Huelsenbeck JP, Willerslev E, Nielsen R (2008). Statistical Assignment of DNA Sequences Using Bayesian Phylogenetics. Syst Biol.

[22] Kearse M (2012). Geneious Basic: An integrated and extendable desktop software platform for the organization and analysis of sequence data. Bioinformatics.

[23] Goldberg CS (2016). Critical considerations for the application of environmental DNA methods to detect aquatic species. Methods Ecol Evol.

[24] Nguyen NH, Smith D, Peay K, Kennedy P (2015). Parsing ecological signal from noise in next generation amplicon sequencing. New Phytol.

[25] Shelton AO (2016). A framework for inferring biological communities from environmental DNA. Ecol Appl.

[26] Brose U, Martinez ND, Williams RJ (2003). Estimating species richness: sensitivity to sample coverage and insensitivity to spatial patterns. Ecology.

[27] Chao A (1984). Nonparametric estimation of the number of classes in a population. Scandinavian Journal of Statistics.

[28] Crist TO, Veech JA (2006). Additive partitioning of rarefaction curves and species–area relationships: unifying α-, β- and γ-diversity with sample size and habitat area. Ecology Letters.

[29] Oksanen, J. F. *et al*. *vegan: Community Ecology Package*. (2016).

[30] Chao A, Chazdon RL, Colwell RK, Shen T-J (2005). A new statistical approach for assessing similarity of species composition with incidence and abundance data. Ecology Letters.

[31] Chiu C-H, Chao A (2016). Estimating and comparing microbial diversity in the presence of sequencing errors. PeerJ.

[32] Chao A (2014). Rarefaction and extrapolation with Hill numbers: a framework for sampling and estimation in species diversity studies. Ecological Monographs.

[33] Hseih, T. C., Ma, K. H. & Chao, A. *iNEXT: iNterpolation and EXTrapolation for species diversity*. (2016).

[34] Trebitz AS, Hoffman JC, Grant GW, Billehus TM, Pilgrim EM (2015). Potential for DNA-based identification of Great Lakes fauna: match and mismatch between taxa inventories and DNA barcode libraries. Scientific Reports.

[35] Wiltshire K, Deveney M (2010). Introduced marine species of South Australia: a review of records and distribution mapping. SARDI Publication No. F2010/000305-1, SARDI Research Report Series No..

[36] Kahle D, Wickham H (2013). ggmap: Spatial Visualization withggplot2. The R Journal.

[37] R Core Team. R: A language and environment for statistical computing. *R Foundation for Statistical Computing, Vienna, Austria*. http://www.R-project.org/ (2013).

