# Author Correction: Effects of sampling effort on biodiversity patterns estimated from environmental DNA metabarcoding surveys

**DOI:** 10.1038/s41598-019-43804-4

**Published:** 2019-10-30

**Authors:** Erin K. Grey, Louis Bernatchez, Phillip Cassey, Kristy Deiner, Marty Deveney, Kimberly L. Howland, Anaïs Lacoursière-Roussel, Sandric Chee Yew Leong, Yiyuan Li, Brett Olds, Michael E. Pfrender, Thomas A. A. Prowse, Mark A. Renshaw, David M. Lodge

**Affiliations:** 1 Division of Science, Mathematics and Technology, Governors State University, 1 University Parkway, University Park, Illinois, 60484 USA; 2 Insitut de Biologie Intégrative et des Systèmes (IBIS), Université Laval, 1030 Avenue de la Médecine, Québec, G1V 0A6 Canada; 3 School of Biological Sciences, University of Adelaide, Adelaide, SA 5005 Australia; 4 Department of Ecology and Evolutionary Biology, Cornell University, 200 Rice Hall, Ithaca, NY 14853 USA; 5 South Australian Aquatic Sciences Centre, 2 Hamra Avenue, West Beach, SA 5024 Australia; 6 Fisheries and Oceans Canada, 501 University Crescent, Winnipeg, Manitoba R3T 2N6 Canada; 7 Tropical Marine Science Institute, National University of Singapore, 18 Kent Ridge Road, S2S Building, Singapore, 119227 Singapore; 8 Department of Biological Sciences, University of Notre Dame, 109b Galvin Life Science Center, Notre Dame, IN 46556 USA; 9 Oceanic Institute, Hawaii Pacific University, 41-202 Kalanianaole Highway, Waimanalo, HI 96795 USA; 10 Atkinson Center for a Sustainable Future, Cornell University, 200 Rice Hall, Ithaca, NY 14853 USA; 11 Environmental Change Initiative, University of Notre Dame, 1400 East Angela Boulevard, Unit 117, South Bend, IN 46617 USA; 12 School of Mathematical Sciences, University of Adelaide, Adelaide, SA 5005 Australia

Correction to: *Scientific Reports* 10.1038/s41598-018-27048-2, published online 11 June 2018

The original version of this Article contained a typographical error in the spelling of the author Kimberly L. Howland, which was incorrectly given as Kimberley L. Howland This has now been corrected in the PDF and HTML versions of the Article, and in the accompanying Supplementary Information files.

