# Supplementary material for: Effects of sampling effort on biodiversity patterns estimated from environmental DNA metabarcoding surveys
Source: Sci Rep. 2018 Jun 11;8:8843. doi: 10.1038/s41598-018-27048-2 (PMC5995838; doi:10.1038/s41598-018-27048-2)

Supplementary Methods:
Environmental DNA Extraction, Amplicon Sequencing, and Bioinformatics Protocols

From manuscript: *Effects of sampling effort on biodiversity patterns estimated from environmental DNA metabarcoding surveys.*

Authors: *Erin K. Grey[1], Louis Bernatchez[2], Phillip Cassey[3], Kristy Deiner[4], Marty Deveney[5], Kimberly L. Howland[6], Anaïs Lacoursière-Roussel[2], Sandric Chee Yew Leong[7], Yiyuan Li[8], Brett Olds[9], Michael E. Pfrender[8], Thomas Prowse[3], Mark A. Renshaw[9], David M. Lodge[4,10]

[1] Division of Science, Mathematics and Technology, Governors State University, 1 University Parkway, University Park, Illinois, 60484
[2] Département de Biologie, Université Laval, 1030 Avenue de la Médecine, Québec (Québec) G1V 0A6, Canada
[3] Ecology and Environmental Science, University of Adelaide, Benhand Building, North Terrace SA 5005, Australia
[4] Department of Ecology and Evolutionary Biology, Cornell University, 200 Rice Hall, Ithaca NY 14853, USA
[5] South Australian Aquatic Sciences Centre, 2 Hamra Avenue, West Beach SA 5024, Australia
[6] Fisheries and Oceans Canada, 501 University Crescent, Winnipeg, Manitoba R3T 2N6, Canada
[7] Tropical Marine Science Institute, National University of Singapore, 18 Kent Ridge Road, S2S Building, Singapore 119227, Singapore
[8] Department of Biological Sciences and Environmental Change Initiative, University of Notre Dame, 109b Galvin Life Science Center, Notre Dame, IN 46556, USA
[9] Oceanic Institute, Hawaii Pacific University, 41-202 Kalanianaole Highway, Waimanalo, HI 96795, USA
[10] Atkinson Center for a Sustainable Future, Cornell University, 200 Rice Hall, Ithaca NY 14853

* Correspondence to egrey@govst.edu

Supplementary Methods 1: Extraction and Amplification Methods for Chicago, Adelaide, and Singapore Samples

Contamination protocol

- Bench-top spaces thoroughly wiped with 10% commercial bleach (0.6% NaClO) prior to use.
- Pipets thoroughly wiped with DNA Away prior to use.
- Single-use filter tips used for all pipetting steps.
- Magnetic stands thoroughly wiped with DNA Away prior to use.
- Single-use razor blades used for all gel extractions; UV tray thoroughly wiped with 10% commercial bleach (0.6% NaClO) prior to and following all gel extractions.
- Strip-tubes (individually capped) used for all PCR steps.
- Entire lab space cleaned weekly, including all bench-top spaces thoroughly wiped with 10% commercial bleach (0.6% NaClO), floor wiped with 10% commercial bleach (0.6% NaClO), and all centrifuges spaces wiped with a combination of DNA Away and 10% commercial bleach (0.6% NaClO).
- Controls (for monitoring of contamination) included in the library prep and carried through to NGS sequencing, regardless of observed PCR amplification:
  a) Field controls (cooler blanks – one per site)
  b) Extraction controls (extraction blanks)
  c) Mock Communities: The mock community for Chicago samples included equals parts DNA from six saltwater fish species (*Amphiprion ocellaris*, *Salaria fasciatus*, *Macropharyngodon negrosensis*, *Ecsenius bicolor*, *Pseduanthias dispar*, and *Centropyge bispinosa*). The mock community for Singapore and Adelaide samples included equal parts DNA from five freshwater fish species (*Umbra limi*, *Thoburnia atripinnis*, *Erimyzon sucetta*, *Notropis topeka*, and *Noturus taylori*).
  d) PCR controls (negative control at 1st stage PCR, plus negative control at 2nd stage PCR)

DNA Extraction Protocol

1) Squeeze membrane against the side of the microtube and add 700µl of chloroform-isoamyl (24:1, Amresco). Vortex tubes for 10 seconds in vertical position.
2) Centrifuge tubes at 15,000g for 5 min. Transfer 500µl aqueous layer to a fresh 1.5mL tube.
3) Add 500µL of ice-cold 100% isopropanol and 250µL of 5 M NaCl to each tube, and precipitate microtubes at -20^0 C for at least one hour.
4) Centrifuge microtubes at 15,000g for 10 minutes to pellet DNA, and then the decant liquid.
5) Add 150 µL of 70% ethanol to each microtube, washing down the inner walls of the tube.
6) Centrifuge tube at 15,000g for 5 minutes and then carefully pour out ethanol.
7) Repeat steps 5 and 6.
8) Dry DNA pellet in vacuufuge at 45^0 C for 15 min. Air-dry until no visible liquid remains.
9) Re-hydrate DNA pellet with 100µL 1X TE Buffer low EDTA warmed to 60^0 C. Heat for 10 minutes in a 55^0 C waterbath.
10) Store at 4^0 C for at least 12 hours before use.

* Please note that primers used in this work contain Illumina specific sequences protected by intellectual property (Oligonucleotide sequences © 2007-2013 Illumina, Inc. All rights reserved. Derivative works created by Illumina customers are authorized for use with Illumina instruments and products only. All other uses are strictly prohibited).

Amplicon Sequencing Protocol

1) PCR-amplify targeted fragment(s) from eDNA sample. The PCR primers have additional oligonucleotides on the 5' end that correspond to part of the Illumina Nextera adaptors.

COI primers:

mLCOI (Leray *et al.* 2013):
5'-TCGTCGGCAGCGTCAGATGTGTATAAGAGACAGGGWACWGGWTGAACWGTWTAYCCYCC-3'
jgHCOI (Geller *et al* 2013):
5'-GTCTCGTGGGCTCGGAGATGTGTATAAGAGACAGTAIACYTCIGGRTGICCRAARAAYCA-3'

COI per sample (50µl) PCR master mix: 30µl H_20, 5µl 10X buffer, 5µl 25mM magnesium, 1.5µl 10mM dNTPs, 1.5µl of 10µM mLCOI, 1.5µl of 10µM jgHCOI, 0.5µl Taq DNA Polymerase (5 PRIME), and 5µl DNA.

COI PCR thermocycling protocol: 16 initial cycles: denaturation for 10s at 95°C, annealing for 30s at 62°C (−1°C per cycle) and extension for 60s at 72°C, followed by 25 cycles at 46°C annealing temperature.

Hadziavdic *et al.* (2014) 18S primers:
18S_574F: 5'TCGTCGGCAGCGTCAGATGTGTATAAGAGACAGGCGGTAATTCCAGCTCCAA3'
18S_952R: 5'GTCTCGTGGGCTCGGAGATGTGTATAAGAGACAGTTGGCAAATGCTTTCGC3'

Hadziavdic 18S per sample (50µl) PCR master mix: 27µl H_20, 10µl 5X HiFi buffer, 1.5µl 50mM magnesium, 1µl 10mM dNTPs, 2.5µl of 10µM primer-F, 2.5µl of 10µM primer-R, 0.5µl Taq DNA Polymerase (iProof, BioRad), and 5µl DNA.

Hadziavdic 18S PCR thermocycling protocol: Initial denaturation at 98°C for 2 minutes; 25 cycles: denaturation at 98°C for 10 seconds, annealing at 55°C for 20 seconds, extension at 72°C for 30 seconds; final extension at 72°C for 10 minutes; and hold indefinitely at 4°C.

2) Run initial (first) PCR amplification on a 2% agarose gel (with 100bp ladder) and band cut amplicon; clean using the QIAquick Gel Extraction Kit (Qiagen) and elute with 25 µl EB. Quantify concentration of DNA with Qubit® HS Assay Kit (Life Technologies). These numbers across amplicons, factoring in the amount combined from each amplicon, is used to estimate the "before enrichment PCR" concentrations.

3) Second round of PCR to attach remaining adaptor sequence (regions that anneal to the flowcell and library specific indices) and increase the amount of DNA:

Nextera ID (forward primer):
5'- AATGATACGGCGACCACCGAGATCTACAC[i5]TCGTCGGCAGCGTC -3'
Nextera ID (reverse primer):
5'- CAAGCAGAAGACGGCATACGAGAT[i7]GTCTCGTGGGCTCGG -3'

* Please note that primers used in this work contain Illumina specific sequences protected by intellectual property (Oligonucleotide sequences © 2007-2013 Illumina, Inc. All rights reserved. Derivative works created by Illumina customers are authorized for use with Illumina instruments and products only. All other uses are strictly prohibited).

i5 indices:
S502 – CTCTCTAT
S503 – TATCCTCT
S504 – AGAGTAGA
S505 – GTAAGGAG
S506 – ACTGCATA
S507 – AAGGAGTA
S508 – CTAAGCCT
S517 – GCGTAAGA

i7 indices:
N701 - TCGCCTTA
N702 - CTAGTACG
N703 - TTCTGCCT
N704 - GCTCAGGA
N705 - AGGAGTCC
N706 - CATGCCTA
N707 - GTAGAGAG
N708 - CCTCTCTG
N709 - AGCGTAGC
N710 - CAGCCTCG
N711 - TGCCTCTT
N712 - TCCTCTAC

2nd PCR per sample (50µl) PCR master mix: 22µl sterile water, 10µl 5X HF buffer, 1.5µl 50mM magnesium, 1µl 10mM dNTPs, 5µl of 10µM Nextera ID (forward primer), 5µl of 10µM Nextera ID (reverse primer), 0.5µl iProof Taq (BioRad), and 5µl DNA (band cut/cleaned from step 2; amplicons combined to produce a similar distribution across amplicons following PCR).
2nd PCR thermocycling protocol: Initial denaturation at 98°C for 2 minutes; 8 cycles: denaturation at 98°C for 10 seconds, annealing at 60°C for 20 seconds, extension at 72°C for 30 seconds; final extension at 72°C for 10 minutes; and hold at 4°C indefinitely.

4) Perform AMPure bead clean-up, 0.8:1 (bead volume : PCR volume) ratio; estimate concentration of DNA with Qubit® HS Assay Kit (Life Technologies). This will be the “after PCR enrichment” concentration compared against “before PCR enrichment” number from step 2, taken into consideration for loading of libraries on the MiSeq.

5) If “before” and “after” Qubit numbers look good, run final QC steps (BioAnalyzer and qPCR), normalize libraries, sequence on the Illumina MiSeq.

* Please note that primers used in this work contain Illumina specific sequences protected by intellectual property (Oligonucleotide sequences © 2007-2013 Illumina, Inc. All rights reserved. Derivative works created by Illumina customers are authorized for use with Illumina instruments and products only. All other uses are strictly prohibited).

Supplementary Methods 2: Extraction and Amplification Methods for Churchill Samples

Contamination Protocol

- Bench-top spaces thoroughly wiped with 10% commercial bleach (0.6% NaClO) prior to use.
- Pipets thoroughly wiped with DNA Away prior to use.
- Single-use filter tips used for all pipetting steps.
- Strip-tubes (individually capped) used for all PCR steps.
- Entire lab space cleaned, including all bench-top spaces thoroughly wiped with 10% commercial bleach (0.6% NaClO) and all centrifuges spaces wiped with a combination of DNA Away and 10% commercial bleach (0.6% NaClO).
- To reduce risk of cross-contamination during sampling and the filtration process, new sterilized gloves, syringes and tweezers were used. They were also exposed to UV for 30 minutes within their original package before putting them in the sampling kits.
- Procedures for eDNA extraction, PCR preparation, and post-PCR steps were all performed in different rooms.
- We developed a one-step dual-indexed PCR approach to eliminate contamination at the second PCR step.
- PCR manipulations were performed in a decontaminated UV hood. The bench space and laboratory tools were bleached and exposed to UV for 30 minutes prior to processing the next port.
- Field negative controls (i.e. 250 mL distilled water) were filtered for every 10 samples. Field extraction negative controls were treated exactly as regular samples and were also sequenced.
- Negative control extractions (950 µL distilled water) were done for each sample batch (i.e. one for each 23 samples). Extraction negative controls were treated exactly as regular samples and were also sequenced.
- Because barcodes were different for each sample, a negative PCR control was done for each sample and primer set. All amplifications were visualized on a 1.5% agarose gel electrophoresis. If positive amplification of the PCR negative control was observed, amplification was redone with a new diluted primer set. Because PCRs negative controls had the same barcodes as samples, they were not sequenced.

DNA Extraction Protocol

1) To isolate and purify eDNA, 30 µl of Proteinase K (Qiagen) was added to the tubes containing the filter and the Longmire buffer. Tubes were vortexed and incubated at 55°C overnight.

2) The filter and lysis solution mixture was centrifuged one minute at 13,000 RPM in a QIAshredder tube. The solution was then transferred within a new tube and 950 µl of the organic phase of phenol chloroform isoamyl alcohol (i.e. PCI, 25:24:1, Sigma P2069) was added. Tubes were hand shaken for five minutes and centrifuged for five minutes at 10,000 RPM. Supernatant was removed into a new tube and 950 µl of Chloroform-Isoamyl alcohol (CI, 24:1) was added to each tube. Tubes were then shaken for five minutes and centrifuged for five minutes at 10,000

* Please note that primers used in this work contain Illumina specific sequences protected by intellectual property (Oligonucleotide sequences © 2007-2013 Illumina, Inc. All rights reserved. Derivative works created by Illumina customers are authorized for use with Illumina instruments and products only. All other uses are strictly prohibited).

RPM. 750 µl of the supernatant was transferred into a new tube and 750 µl of ice cold isopropanol and 375 µl of room temperature 5M NaCl were added to each tube and left overnight at -4°C. Tubes were centrifuged for 20 minutes at 13,000 RPM and isopropanol was then carefully poured off. 1,500 µl of cold Ethanol 70% was added and centrifuged for 20 minutes at 13,000 RPM. Ethanol was then carefully poured off and tubes were air dried with lid open in a laminar flow hood for 15 minutes.

3) DNA was resuspended in 80 µl sterilized water (diH$_2$0), placed in an incubator at 55°C for ten minutes and at 4°C overnight to dissolve DNA. The extracted DNA was then frozen at -20 °C until amplification.

Amplicon Sequencing Protocol

DNA amplifications were performed in a one-step dual-indexed PCR approach specifically designed for Illumina instruments by the "Plate-forme d'Analyses Génomiques" (IBIS, Université Laval). The primers were tailed on the 5' end with the Illumina Nextera adaptors*. The following adaptor sequence (including regions that anneal to the flowcell, library specific indices, and sequencing primer binding sites) and oligonucleotide primer sequences were used for amplification:

COI primers:
mLCOI (Leray *et al.* 2013)**:**
5'-AATGATACGGCGACCACCGAGATCTACAC-[INDEX]-TCGTCGGCAGCGTCAGATGTGTATAAGAGACAG-GGWACWGGWTGAACWGTWTAYCCYCC-3'
jgHCOI (Geller *et al.* 2013)**:**
5'- CAAGCAGAAGACGGCATACGAGAT-[INDEX]-GTCTCGTGGGCTCGGAGATGTGTATAAGAGACAG-TAIACYTCIGGRTGICCRAARAAYCA-3'

Hadziavdic *et al.* (2014) 18S primers:
18S_574F: 5'-AATGATACGGCGACCACCGAGATCTACAC-[INDEX]-TCGTCGGCAGCGTCAGATGTGTATAAGAGACAG-GCGGTAATTCCAGCTCCAA-3'
18S_952R: 5'- CAAGCAGAAGACGGCATACGAGAT-[INDEX]-GTCTCGTGGGCTCGGAGATGTGTATAAGAGACAG-TTGGCAAATGCTTTCGC-3'

1) Three PCR replicates were done for each sample and each primer set. The final reaction volume for each PCR replicate was 24 µL; including 12.5. µl Qiagen Multiplex Mastermix, 6.5 µl diH$_2$0, 1 µl of each primer (10µM), and 3.0 µL of DNA. For all samples, the PCR mixture was denatured at 95°C for 15 min, followed by 35 cycles (94°C for 30 s, 54°C for 90 s and 72°C for 60s) and a final elongation at 72°C for 10 min. Products of the three aliquots were pooled for each sample.

2) Pooled products were purified using Axygen PCR clean up kit following the manufacture's recommended protocol. Libraries were quantified by AccuClear Ultra High Sensitivity dsDNA Quantitation Kit using the TECAN Spark 10M Reader for each sample and were pooled in equal molar concentrations to maximize equal sequence depth per sample location (150 ng total).

* Please note that primers used in this work contain Illumina specific sequences protected by intellectual property (Oligonucleotide sequences © 2007-2013 Illumina, Inc. All rights reserved. Derivative works created by Illumina customers are authorized for use with Illumina instruments and products only. All other uses are strictly prohibited).

When Quant-iT PicoGreen (Life Technologies) did not detect any DNA, 22.0 µL PCR mixtures were mixed nonetheless.

3) Sequencing was carried out using an Illumina MiSeq (Illumina, San Diego, USA) at IBIS using a paired-end MiSeq Reagent Kit V3 (Illumina, San Diego, USA; sequence length = 300bp) and following the manufacturer's instructions. For sequencing, the amplicon pool was diluted to 4 nM with molecular grade water, denatured and then sequenced at 10 pM following manufacturer's instructions.

Literature cited:

Geller, J., C. Meyer, M. Parker, and H. Hawk (2013) Redesign of PCR primers for mitochondrial cytochrome c oxidase subunit I for marine invertebrates and application in all-taxa biotic surveys. *Molecular Ecology Resources* 13(5): 851-861

Hadziavdic K, Lekang K, Lanzen A, Jonassen I, Thompson EM, Troedsson C (2014) Characterization of the 18S rRNA gene for designing universal Eukaryote specific primers. PLoS ONE 9: e87624.

Leray M, Yang JY, Meyer CP, Mills SC, Agudelo N, Ranwez V, Boehm JT, Machida RJ (2013) A new versatile primer set targeting a short fragment of the mitochondrial COI region for metabarcoding metazoan diversity: application for characterizing coral reef fish gut contents. Frontiers in Zoology 10: 34.

Renshaw, M. A., Olds, B. P., Jerde, C. L., McVeigh, M. M., & Lodge, D. M. (2015). The room temperature preservation of filtered environmental DNA samples and assimilation into a phenol–chloroform–isoamyl alcohol DNA extraction. *Molecular ecology resources*, *15*(1), 168-176.

* Please note that primers used in this work contain Illumina specific sequences protected by intellectual property (Oligonucleotide sequences © 2007-2013 Illumina, Inc. All rights reserved. Derivative works created by Illumina customers are authorized for use with Illumina instruments and products only. All other uses are strictly prohibited).

Supplementary Methods 3: Bioinformatics Flowchart for All Samples

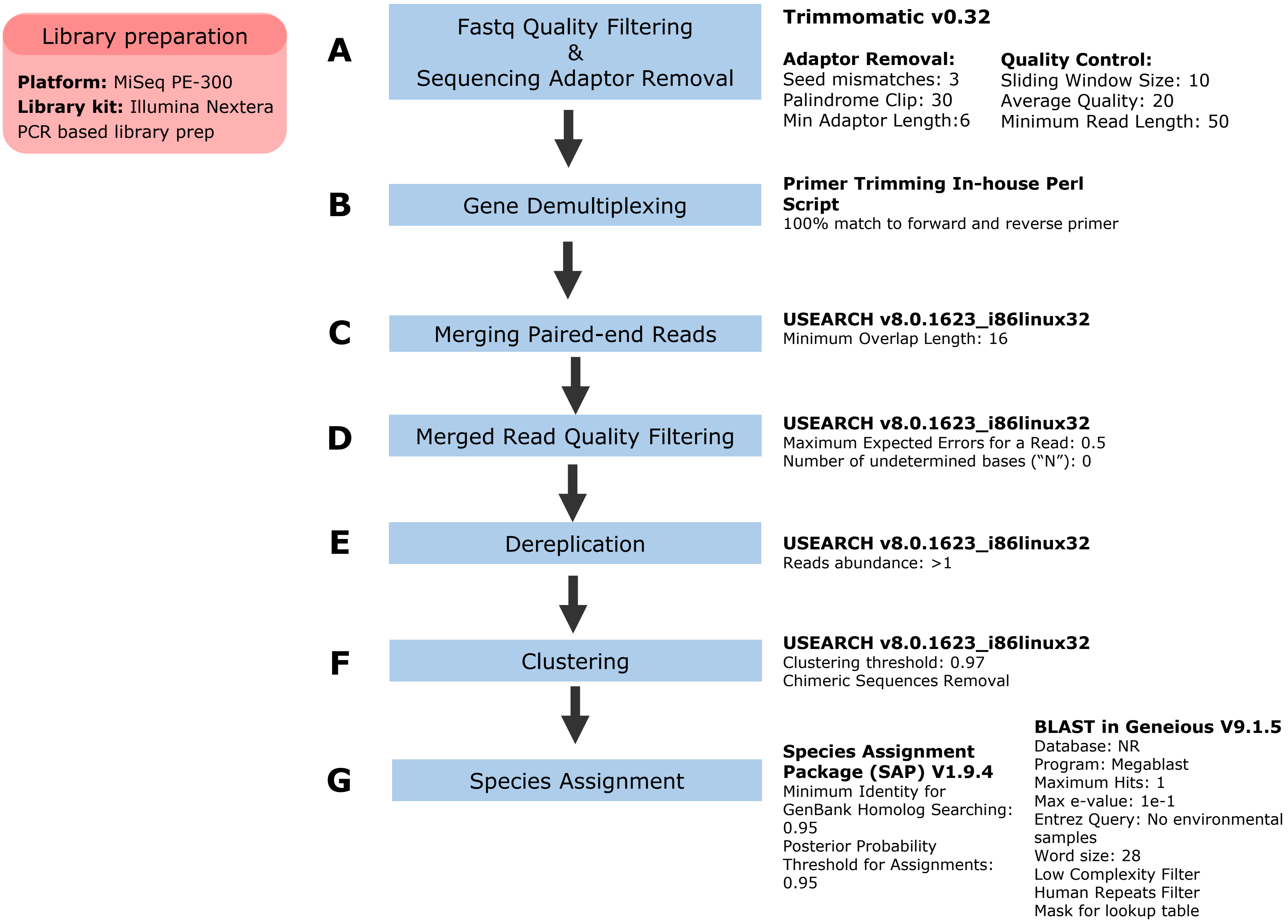

Supplement: Supplementary file 1 — Supplementary Methods [file 41598_2018_27048_MOESM1_ESM.pdf]
