# Supplementary material for: Effects of sampling effort on biodiversity patterns estimated from environmental DNA metabarcoding surveys
Source: Sci Rep. 2018 Jun 11;8:8843. doi: 10.1038/s41598-018-27048-2 (PMC5995838; doi:10.1038/s41598-018-27048-2)
Supplement: Supplementary file 2 — Supplementary Information [file 41598_2018_27048_MOESM2_ESM.pdf]

# Supplementary Information

From manuscript: *Effects of sampling effort on biodiversity patterns estimated from environmental DNA metabarcoding surveys.*

Authors: *Erin K. Grey[1], Louis Bernatchez[2], Phillip Cassey[3], Kristy Deiner[4], Marty Deveney[5], Kimberly L. Howland[6], Anaïs Lacoursière-Roussel[2], Sandric Chee Yew Leong[7], Yiyuan Li[8], Brett Olds[9], Michael E. Pfrender[8], Thomas Prowse[3], Mark A. Renshaw[9], David M. Lodge[4,10]

[1] Division of Science, Mathematics and Technology, Governors State University, 1 University Parkway, University Park, Illinois, 60484
[2] Département de Biologie, Université Laval, 1030 Avenue de la Médecine, Québec (Québec) G1V 0A6, Canada
[3] Ecology and Environmental Science, University of Adelaide, Benhand Building, North Terrace SA 5005, Australia
[4] Department of Ecology and Evolutionary Biology, Cornell University, 200 Rice Hall, Ithaca NY 14853, USA
[5] South Australian Aquatic Sciences Centre, 2 Hamra Avenue, West Beach SA 5024, Australia
[6] Fisheries and Oceans Canada, 501 University Crescent, Winnipeg, Manitoba R3T 2N6, Canada
[7] Tropical Marine Science Institute, National University of Singapore, 18 Kent Ridge Road, S2S Building, Singapore 119227, Singapore
[8] Department of Biological Sciences and Environmental Change Initiative, University of Notre Dame, 109b Galvin Life Science Center, Notre Dame, IN 46556, USA
[9] Oceanic Institute, Hawaii Pacific University, 41-202 Kalanianaole Highway, Waimanalo, HI 96795, USA
[10] Atkinson Center for a Sustainable Future, Cornell University, 200 Rice Hall, Ithaca NY 14853

*Correspondence to egrey@govst.edu

**Supplementary Table S1.** Sequencing summaries by sample and site. "Quality Reads" = number of reads after trimming adaptors and primers, merging paired-end reads, and quality filtering (Supplementary Methods: Bioinformatics Steps A-E). "Quality MOTUs" = number of MOTUs after clustering (Bioinformatics Step F). "Filtered Reads" and "Filtered MOTUs" = number of reads and MOTUs after removing contamination controls, respectively. "Metazoan Reads" and "Metazoan MOTUs" = number of reads or MOTUs assigned to a metazoan phylum, respectively.

| Bar-code | Sample ID | Site | Quality Reads | Quality MOTUs | Filtered Reads | Filtered MOTUs | Metazoan Reads | Metazoan MOTUs |
|---|---|---|---|---|---|---|---|---|
| 18S | out34_1 | Chicago | 112,152 | 484 | 111,736 | 464 | 11,543 | 23 |
| 18S | out34_10 | Chicago | 372,393 | 708 | 371,801 | 684 | 42,471 | 35 |
| 18S | out34_11 | Chicago | 97,856 | 451 | 97,480 | 436 | 2,406 | 22 |
| 18S | out34_12 | Chicago | 183,205 | 539 | 182,763 | 522 | 55,181 | 31 |
| 18S | out34_13 | Chicago | 241,897 | 636 | 241,070 | 617 | 5,726 | 18 |
| 18S | out34_14 | Chicago | 213,676 | 501 | 213,309 | 484 | 95,989 | 17 |
| 18S | out34_15 | Chicago | 85,577 | 341 | 85,170 | 324 | 53,719 | 22 |
| 18S | out34_16 | Chicago | 296,332 | 662 | 295,842 | 637 | 16,573 | 26 |
| 18S | out34_17 | Chicago | 360,491 | 755 | 359,813 | 728 | 30,128 | 27 |
| 18S | out34_18 | Chicago | 352,117 | 713 | 351,610 | 694 | 86,225 | 38 |
| 18S | out34_19 | Chicago | 266,040 | 629 | 265,570 | 610 | 30,899 | 32 |
| 18S | out34_2 | Chicago | 231,713 | 545 | 231,302 | 520 | 18,936 | 30 |
| 18S | out34_20 | Chicago | 345,626 | 778 | 344,874 | 754 | 29,947 | 36 |
| 18S | out34_3 | Chicago | 177,908 | 522 | 177,496 | 504 | 16,827 | 25 |
| 18S | out34_4 | Chicago | 114,906 | 485 | 114,526 | 466 | 8,827 | 19 |
| 18S | out34_5 | Chicago | 237,762 | 647 | 237,362 | 633 | 8,526 | 27 |
| 18S | out34_6 | Chicago | 286,419 | 544 | 286,026 | 528 | 19,682 | 26 |
| 18S | out34_7 | Chicago | 328,132 | 598 | 327,739 | 585 | 11,696 | 23 |
| 18S | out34_8 | Chicago | 344,749 | 647 | 344,268 | 630 | 26,836 | 28 |
| 18S | out34_9 | Chicago | 416,771 | 661 | 416,331 | 644 | 38,961 | 30 |
| **18S** | **ALL** | **Chicago** | **5,065,722** | **1,788** | **5,056,088** | **1,758** | **611,098** | **100** |
| 18S | CH_30 | Churchill | 35,429 | 638 | 28,884 | 595 | 12,756 | 38 |
| 18S | CH_31 | Churchill | 35,500 | 798 | 29,054 | 733 | 9,417 | 47 |
| 18S | CH_32 | Churchill | 39,566 | 987 | 32,851 | 930 | 10,916 | 54 |
| 18S | CH_33 | Churchill | 43,162 | 1,197 | 40,568 | 1,148 | 1,240 | 63 |
| 18S | CH_34 | Churchill | 24,899 | 582 | 18,928 | 540 | 11,135 | 38 |
| 18S | CH_35 | Churchill | 41,243 | 976 | 37,127 | 927 | 1,813 | 67 |
| 18S | CH_36 | Churchill | 5,702 | 332 | 2,345 | 292 | 185 | 22 |
| 18S | CH_37 | Churchill | 33,378 | 810 | 26,466 | 747 | 13,579 | 47 |
| 18S | CH_38 | Churchill | 42,308 | 1,130 | 35,695 | 1,073 | 4,137 | 52 |
| 18S | CH_39 | Churchill | 38,169 | 976 | 31,317 | 926 | 8,622 | 60 |
| 18S | CH_40 | Churchill | 36,528 | 1,024 | 29,480 | 963 | 8,078 | 58 |
| 18S | CH_43 | Churchill | 37,056 | 797 | 33,185 | 750 | 16,670 | 49 |
| 18S | CH_44 | Churchill | 33,140 | 985 | 31,069 | 935 | 5,310 | 52 |
| 18S | CH_45 | Churchill | 44,939 | 1,047 | 42,938 | 996 | 3,054 | 49 |
| 18S | CH_46 | Churchill | 37,066 | 907 | 30,217 | 858 | 5,024 | 54 |
| 18S | CH_47 | Churchill | 43,338 | 1,014 | 40,234 | 961 | 9,165 | 55 |
| 18S | CH_48 | Churchill | 46,807 | 1,155 | 44,473 | 1,108 | 2,406 | 63 |
| 18S | CH_49 | Churchill | 39,750 | 933 | 35,916 | 880 | 1,049 | 48 |
| 18S | CH_50 | Churchill | 39,277 | 725 | 32,788 | 685 | 13,922 | 46 |
| 18S | CH_70 | Churchill | 36,784 | 587 | 30,525 | 550 | 14,585 | 29 |
| **18S** | **ALL** | **Churchill** | **734,041** | **2,390** | **634,060** | **2,310** | **153,063** | **172** |

| Bar-code | Sample ID | Site | Quality Reads | Quality MOTUs | Filtered Reads | Filtered MOTUs | Metazoan Reads | Metazoan MOTUs |
|---|---|---|---|---|---|---|---|---|
| 18S | out26_01 | Singapore Yacht | 710,100 | 796 | 708,378 | 786 | 146,731 | 107 |
| 18S | out26_02 | Singapore Yacht | 614,187 | 952 | 612,457 | 942 | 278,600 | 134 |
| 18S | out26_03 | Singapore Yacht | 526,498 | 813 | 524,907 | 806 | 141,200 | 117 |
| 18S | out26_04 | Singapore Yacht | 543,833 | 738 | 542,232 | 728 | 412,363 | 116 |
| 18S | out26_05 | Singapore Yacht | 601,939 | 787 | 600,491 | 775 | 131,082 | 98 |
| 18S | out26_06 | Singapore Yacht | 393,673 | 794 | 392,285 | 783 | 129,051 | 121 |
| 18S | out35_1 | Singapore Yacht | 99,108 | 559 | 98,387 | 530 | 74,476 | 118 |
| 18S | out35_10 | Singapore Yacht | 187,180 | 804 | 186,078 | 782 | 132,189 | 127 |
| 18S | out35_11 | Singapore Yacht | 109,744 | 617 | 109,125 | 593 | 96,007 | 124 |
| 18S | out35_12 | Singapore Yacht | 246,212 | 571 | 245,292 | 545 | 233,286 | 125 |
| 18S | out35_13 | Singapore Yacht | 211,161 | 805 | 209,652 | 770 | 164,178 | 157 |
| 18S | out35_14 | Singapore Yacht | 338,953 | 856 | 337,360 | 837 | 280,014 | 142 |
| 18S | out35_2 | Singapore Yacht | 158,289 | 639 | 157,220 | 614 | 100,291 | 94 |
| 18S | out35_3 | Singapore Yacht | 148,819 | 663 | 148,044 | 635 | 23,088 | 95 |
| 18S | out35_4 | Singapore Yacht | 479,148 | 974 | 477,544 | 952 | 190,589 | 154 |
| 18S | out35_5 | Singapore Yacht | 114,730 | 793 | 113,706 | 777 | 47,513 | 130 |
| 18S | out35_6 | Singapore Yacht | 310,293 | 837 | 309,073 | 815 | 258,138 | 148 |
| 18S | out35_7 | Singapore Yacht | 257,121 | 763 | 255,675 | 743 | 129,575 | 121 |
| 18S | out35_8 | Singapore Yacht | 104,614 | 575 | 103,955 | 545 | 62,644 | 88 |
| 18S | out35_9 | Singapore Yacht | 92,557 | 517 | 92,136 | 487 | 83,720 | 120 |
| **18S** | **ALL** | **Singapore Yacht** | **6,248,159** | **2,555** | **6,223,997** | **2,527** | **3,114,735** | **419** |
| 18S | out26_07 | Singapore Woodlands | 170,998 | 488 | 169,748 | 480 | 18,951 | 28 |
| 18S | out26_08 | Singapore Woodlands | 263,965 | 453 | 262,975 | 437 | 8,172 | 36 |
| 18S | out26_09 | Singapore Woodlands | 234,551 | 459 | 233,262 | 452 | 4,332 | 29 |
| 18S | out26_10 | Singapore Woodlands | 257,302 | 502 | 256,010 | 490 | 7,495 | 26 |
| 18S | out26_11 | Singapore Woodlands | 218,449 | 491 | 217,193 | 479 | 12,406 | 33 |
| 18S | out26_12 | Singapore Woodlands | 200,661 | 464 | 199,501 | 454 | 13,567 | 30 |
| 18S | out26_13 | Singapore Woodlands | 243,872 | 497 | 242,752 | 480 | 4,985 | 28 |
| 18S | out26_14 | Singapore Woodlands | 220,369 | 474 | 219,445 | 463 | 10,676 | 23 |
| 18S | out35_15 | Singapore Woodlands | 321,395 | 510 | 320,112 | 499 | 22,321 | 41 |
| 18S | out35_16 | Singapore Woodlands | 293,434 | 483 | 292,132 | 469 | 6,169 | 25 |
| 18S | out35_17 | Singapore Woodlands | 116,547 | 416 | 115,559 | 395 | 1,296 | 28 |
| 18S | out35_18 | Singapore Woodlands | 137,119 | 398 | 136,628 | 379 | 20,507 | 34 |
| 18S | out35_19 | Singapore Woodlands | 132,039 | 428 | 130,868 | 410 | 30,252 | 36 |
| 18S | out35_20 | Singapore Woodlands | 187,292 | 493 | 186,250 | 475 | 11,632 | 36 |
| 18S | out35_21 | Singapore Woodlands | 186,710 | 489 | 185,489 | 472 | 35,012 | 46 |
| 18S | out35_22 | Singapore Woodlands | 50,148 | 347 | 49,087 | 335 | 13,103 | 34 |
| 18S | out35_23 | Singapore Woodlands | 133,436 | 410 | 132,782 | 397 | 28,206 | 50 |
| 18S | out35_24 | Singapore Woodlands | 239,709 | 484 | 238,481 | 469 | 83,716 | 40 |
| 18S | out35_25 | Singapore Woodlands | 120,000 | 410 | 119,213 | 397 | 14,502 | 38 |
| 18S | out35_26 | Singapore Woodlands | 225,137 | 461 | 223,888 | 449 | 31,997 | 40 |
| **18S** | **ALL** | **Singapore Woodlands** | **3,953,133** | **1,288** | **3,931,375** | **1,267** | **379,297** | **121** |
| **18S** | **ALL** | **Singapore All Sites** | **10,201,292** | **3,114** | **10,155,372** | **3,088** | **3,494,032** | **467** |
| 18S | out29_59 | Adelaide Container Channel | 232,612 | 929 | 230,954 | 906 | 120,748 | 120 |
| 18S | out29_60 | Adelaide Container Channel | 247,967 | 726 | 246,370 | 702 | 193,117 | 96 |
| 18S | out29_61 | Adelaide Container Channel | 237,491 | 788 | 235,983 | 769 | 170,309 | 106 |

| Bar-code | Sample ID | Site | Quality Reads | Quality MOTUs | Filtered Reads | Filtered MOTUs | Metazoan Reads | Metazoan MOTUs |
|---|---|---|---|---|---|---|---|---|
| 18S | out29_62 | Adelaide Container Channel | 308,303 | 864 | 306,828 | 844 | 194,122 | 103 |
| 18S | out29_63 | Adelaide Container Channel | 300,986 | 923 | 299,426 | 897 | 169,492 | 127 |
| 18S | out29_64 | Adelaide Container Channel | 58,367 | 562 | 56,999 | 542 | 26,464 | 54 |
| 18S | out29_65 | Adelaide Container Channel | 262,156 | 916 | 260,614 | 894 | 139,165 | 90 |
| 18S | out29_66 | Adelaide Container Channel | 264,557 | 871 | 262,999 | 839 | 155,178 | 80 |
| 18S | out29_67 | Adelaide Container Channel | 281,811 | 877 | 280,351 | 854 | 171,224 | 98 |
| **18S** | **ALL** | **Adelaide Container Channel** | **2,194,250** | **1,909** | **2,180,524** | **1,876** | **1,339,819** | **274** |
| 18S | out29_78 | Adelaide Container Dock 1 | 201,113 | 814 | 199,482 | 795 | 106,377 | 89 |
| 18S | out29_79 | Adelaide Container Dock 1 | 234,207 | 997 | 232,657 | 974 | 77,647 | 100 |
| 18S | out29_80 | Adelaide Container Dock 1 | 187,289 | 883 | 185,758 | 854 | 63,856 | 94 |
| 18S | out29_81 | Adelaide Container Dock 1 | 272,886 | 792 | 271,445 | 759 | 190,675 | 74 |
| 18S | out29_82 | Adelaide Container Dock 1 | 201,198 | 789 | 199,733 | 769 | 121,166 | 100 |
| 18S | out29_83 | Adelaide Container Dock 1 | 226,518 | 920 | 224,817 | 899 | 109,594 | 116 |
| 18S | out29_84 | Adelaide Container Dock 1 | 206,084 | 599 | 204,708 | 579 | 166,747 | 89 |
| 18S | out29_85 | Adelaide Container Dock 1 | 216,684 | 871 | 214,470 | 845 | 129,799 | 116 |
| 18S | out29_86 | Adelaide Container Dock 1 | 210,148 | 962 | 208,585 | 937 | 45,389 | 104 |
| **18S** | **ALL** | **Adelaide Container Dock 1** | **1,956,127** | **1,998** | **1,941,655** | **1,963** | **1,011,250** | **324** |
| 18S | out29_77 | Adelaide Container Dock 2 | 293,558 | 1,170 | 291,918 | 1,141 | 47,951 | 95 |
| 18S | out29_87 | Adelaide Container Dock 2 | 234,205 | 977 | 232,635 | 959 | 119,395 | 105 |
| 18S | out29_88 | Adelaide Container Dock 2 | 178,168 | 962 | 176,682 | 937 | 44,267 | 82 |
| 18S | out29_89 | Adelaide Container Dock 2 | 199,365 | 932 | 197,877 | 906 | 53,748 | 59 |
| 18S | out29_90 | Adelaide Container Dock 2 | 178,810 | 908 | 177,381 | 888 | 62,571 | 70 |
| 18S | out29_91 | Adelaide Container Dock 2 | 232,936 | 897 | 231,361 | 875 | 137,374 | 93 |
| 18S | out29_92 | Adelaide Container Dock 2 | 188,463 | 946 | 186,947 | 917 | 49,728 | 77 |
| 18S | out29_93 | Adelaide Container Dock 2 | 180,806 | 937 | 179,265 | 911 | 62,441 | 76 |
| 18S | out29_94 | Adelaide Container Dock 2 | 195,905 | 927 | 194,194 | 903 | 65,559 | 76 |
| 18S | out29_95 | Adelaide Container Dock 2 | 203,910 | 965 | 202,336 | 946 | 77,985 | 64 |
| **18S** | **ALL** | **Adelaide Container Dock 2** | **2,086,126** | **2,231** | **2,070,596** | **2,196** | **721,019** | **255** |
| 18S | out29_10 | Adelaide Fuel Channel | 134,341 | 791 | 132,673 | 771 | 50,408 | 56 |
| 18S | out29_11 | Adelaide Fuel Channel | 139,964 | 759 | 138,396 | 737 | 62,767 | 71 |
| 18S | out29_12 | Adelaide Fuel Channel | 129,847 | 780 | 128,288 | 764 | 53,742 | 68 |
| 18S | out29_13 | Adelaide Fuel Channel | 138,707 | 739 | 137,078 | 715 | 65,074 | 49 |
| 18S | out29_14 | Adelaide Fuel Channel | 223,318 | 941 | 221,614 | 919 | 58,547 | 65 |
| 18S | out29_16 | Adelaide Fuel Channel | 163,952 | 790 | 162,376 | 772 | 69,046 | 58 |
| 18S | out29_17 | Adelaide Fuel Channel | 176,837 | 792 | 175,218 | 777 | 78,816 | 59 |
| 18S | out29_18 | Adelaide Fuel Channel | 235,138 | 867 | 233,544 | 849 | 128,289 | 86 |
| 18S | out29_19 | Adelaide Fuel Channel | 190,720 | 877 | 189,069 | 855 | 75,926 | 66 |
| **18S** | **ALL** | **Adelaide Fuel Channel** | **1,532,824** | **1,694** | **1,518,256** | **1,659** | **642,615** | **184** |
| 18S | out29_1 | Adelaide Fuel Dock | 140,578 | 836 | 139,081 | 808 | 56,093 | 72 |
| 18S | out29_2 | Adelaide Fuel Dock | 157,400 | 840 | 155,855 | 819 | 67,786 | 92 |
| 18S | out29_3 | Adelaide Fuel Dock | 202,301 | 884 | 200,740 | 862 | 53,840 | 53 |
| 18S | out29_4 | Adelaide Fuel Dock | 222,385 | 756 | 220,856 | 742 | 131,009 | 77 |
| 18S | out29_5 | Adelaide Fuel Dock | 179,635 | 834 | 178,071 | 819 | 66,238 | 69 |
| 18S | out29_6 | Adelaide Fuel Dock | 180,599 | 784 | 178,977 | 767 | 88,142 | 74 |
| 18S | out29_7 | Adelaide Fuel Dock | 180,265 | 824 | 178,684 | 802 | 69,713 | 72 |
| 18S | out29_76 | Adelaide Fuel Dock | 290,938 | 1,064 | 289,279 | 1,036 | 125,316 | 102 |

| Bar-code | Sample ID | Site | Quality Reads | Quality MOTUs | Filtered Reads | Filtered MOTUs | Metazoan Reads | Metazoan MOTUs |
|---|---|---|---|---|---|---|---|---|
| 18S | out29_8 | Adelaide Fuel Dock | 138,045 | 719 | 136,584 | 698 | 39,353 | 57 |
| 18S | out29_9 | Adelaide Fuel Dock | 177,901 | 890 | 176,281 | 860 | 60,184 | 76 |
| **18S** | **ALL** | **Adelaide Fuel Dock** | **1,870,047** | **1,892** | **1,854,408** | **1,854** | **757,674** | **223** |
| 18S | out29_49 | Adelaide Marina Channel | 430,581 | 839 | 428,944 | 808 | 341,688 | 84 |
| 18S | out29_50 | Adelaide Marina Channel | 193,133 | 907 | 191,516 | 892 | 76,710 | 83 |
| 18S | out29_51 | Adelaide Marina Channel | 462,499 | 1,073 | 460,698 | 1,042 | 264,013 | 119 |
| 18S | out29_52 | Adelaide Marina Channel | 160,091 | 786 | 158,504 | 758 | 91,090 | 98 |
| 18S | out29_53 | Adelaide Marina Channel | 248,272 | 875 | 246,592 | 851 | 136,267 | 80 |
| 18S | out29_54 | Adelaide Marina Channel | 208,713 | 834 | 207,042 | 810 | 125,142 | 98 |
| 18S | out29_55 | Adelaide Marina Channel | 268,796 | 922 | 267,171 | 895 | 151,492 | 89 |
| 18S | out29_56 | Adelaide Marina Channel | 288,131 | 945 | 286,544 | 922 | 151,704 | 90 |
| 18S | out29_57 | Adelaide Marina Channel | 309,345 | 1,000 | 307,530 | 980 | 163,569 | 94 |
| 18S | out29_75 | Adelaide Marina Channel | 259,823 | 924 | 258,145 | 904 | 112,064 | 74 |
| **18S** | **ALL** | **Adelaide Marina Channel** | **2,829,384** | **2,084** | **2,812,686** | **2,043** | **1,613,739** | **285** |
| 18S | out29_39 | Adelaide Marina Dock | 170,564 | 571 | 169,138 | 554 | 125,488 | 61 |
| 18S | out29_40 | Adelaide Marina Dock | 90,042 | 466 | 88,643 | 450 | 73,924 | 66 |
| 18S | out29_41 | Adelaide Marina Dock | 173,455 | 595 | 172,025 | 572 | 135,102 | 66 |
| 18S | out29_42 | Adelaide Marina Dock | 168,384 | 668 | 166,902 | 649 | 112,110 | 61 |
| 18S | out29_43 | Adelaide Marina Dock | 172,680 | 702 | 171,151 | 684 | 123,599 | 90 |
| 18S | out29_45 | Adelaide Marina Dock | 183,367 | 733 | 181,761 | 713 | 132,650 | 94 |
| 18S | out29_46 | Adelaide Marina Dock | 266,956 | 841 | 265,370 | 822 | 183,898 | 105 |
| 18S | out29_47 | Adelaide Marina Dock | 186,420 | 666 | 184,871 | 637 | 148,147 | 71 |
| 18S | out29_48 | Adelaide Marina Dock | 173,421 | 700 | 171,875 | 683 | 128,867 | 74 |
| **18S** | **ALL** | **Adelaide Marina Dock** | **1,585,289** | **1,546** | **1,571,736** | **1,514** | **1,163,785** | **222** |
| **18S** | **ALL** | **Adelaide All Sites** | **14,054,047** | **3,930** | **13,949,861** | **3,882** | **7,249,901** | **608** |
| COI | out34_1 | Chicago | 242,966 | 380 | 42,574 | 340 | 33,529 | 124 |
| COI | out34_10 | Chicago | 24,906 | 212 | 7,392 | 140 | 5,202 | 54 |
| COI | out34_11 | Chicago | 233,919 | 428 | 55,855 | 388 | 40,777 | 136 |
| COI | out34_12 | Chicago | 224,801 | 421 | 48,581 | 377 | 33,445 | 129 |
| COI | out34_13 | Chicago | 199,972 | 326 | 16,316 | 262 | 12,851 | 96 |
| COI | out34_14 | Chicago | 176,951 | 201 | 94,147 | 128 | 92,281 | 41 |
| COI | out34_15 | Chicago | 36,079 | 125 | 7,878 | 54 | 7,597 | 24 |
| COI | out34_16 | Chicago | 113,085 | 202 | 1,272 | 129 | 530 | 57 |
| COI | out34_17 | Chicago | 80,020 | 280 | 4,085 | 203 | 773 | 69 |
| COI | out34_18 | Chicago | 51,436 | 205 | 10,120 | 140 | 7,640 | 51 |
| COI | out34_19 | Chicago | 104,141 | 255 | 3,669 | 181 | 896 | 61 |
| COI | out34_2 | Chicago | 180,992 | 372 | 21,190 | 311 | 7,507 | 97 |
| COI | out34_20 | Chicago | 105,143 | 320 | 6,171 | 247 | 1,074 | 98 |
| COI | out34_3 | Chicago | 115,174 | 334 | 16,456 | 259 | 3,426 | 80 |
| COI | out34_4 | Chicago | 79,851 | 275 | 4,107 | 194 | 780 | 63 |
| COI | out34_5 | Chicago | 79,552 | 265 | 7,535 | 196 | 749 | 63 |
| COI | out34_6 | Chicago | 66,608 | 279 | 10,358 | 207 | 1,592 | 63 |
| COI | out34_7 | Chicago | 61,797 | 258 | 9,052 | 172 | 3,190 | 50 |
| COI | out34_8 | Chicago | 42,352 | 263 | 7,995 | 195 | 878 | 63 |
| COI | out34_9 | Chicago | 37,091 | 228 | 7,822 | 169 | 1,069 | 55 |
| **COI** | **ALL** | **Chicago** | **2,256,836** | **892** | **382,575** | **854** | **255,786** | **333** |

| Bar-code | Sample ID | Site | Quality Reads | Quality MOTUs | Filtered Reads | Filtered MOTUs | Metazoan Reads | Metazoan MOTUs |
|---|---|---|---|---|---|---|---|---|
| COI | CH_30 | Churchill | 50,613 | 2,311 | 50,229 | 2,287 | 16,314 | 782 |
| COI | CH_31 | Churchill | 39,340 | 2,129 | 38,987 | 2,104 | 14,378 | 715 |
| COI | CH_32 | Churchill | 48,548 | 2,471 | 48,099 | 2,442 | 12,935 | 829 |
| COI | CH_33 | Churchill | 49,871 | 2,574 | 49,401 | 2,541 | 13,578 | 847 |
| COI | CH_34 | Churchill | 36,527 | 1,803 | 36,185 | 1,774 | 11,179 | 603 |
| COI | CH_35 | Churchill | 37,001 | 2,429 | 36,617 | 2,396 | 10,466 | 730 |
| COI | CH_36 | Churchill | 14,568 | 1,707 | 14,278 | 1,681 | 3,902 | 503 |
| COI | CH_37 | Churchill | 32,207 | 2,320 | 31,835 | 2,294 | 7,695 | 714 |
| COI | CH_38 | Churchill | 34,940 | 2,334 | 34,588 | 2,301 | 8,290 | 715 |
| COI | CH_39 | Churchill | 46,564 | 2,463 | 46,165 | 2,431 | 11,718 | 794 |
| COI | CH_40 | Churchill | 55,590 | 2,840 | 55,072 | 2,805 | 16,105 | 884 |
| COI | CH_43 | Churchill | 42,005 | 2,078 | 41,602 | 2,046 | 15,562 | 658 |
| COI | CH_44 | Churchill | 37,301 | 2,220 | 36,950 | 2,193 | 10,644 | 701 |
| COI | CH_45 | Churchill | 39,880 | 2,247 | 39,475 | 2,223 | 10,066 | 674 |
| COI | CH_46 | Churchill | 40,052 | 2,242 | 39,671 | 2,222 | 11,275 | 682 |
| COI | CH_47 | Churchill | 39,257 | 2,060 | 38,907 | 2,036 | 10,715 | 633 |
| COI | CH_48 | Churchill | 35,945 | 2,154 | 35,565 | 2,125 | 9,483 | 683 |
| COI | CH_49 | Churchill | 30,098 | 1,907 | 29,767 | 1,885 | 7,670 | 598 |
| COI | CH_50 | Churchill | 45,652 | 2,243 | 45,269 | 2,209 | 11,985 | 726 |
| COI | CH_70 | Churchill | 47,023 | 2,258 | 46,626 | 2,231 | 13,842 | 728 |
| **COI** | **ALL** | **Churchill** | **802,982** | **5,645** | **795,288** | **5,600** | **227,802** | **1,937** |
| COI | out35_1 | Singapore Yacht | 79,376 | 522 | 79,201 | 517 | 59,156 | 216 |
| COI | out35_10 | Singapore Yacht | 56,528 | 564 | 56,340 | 550 | 33,877 | 224 |
| COI | out35_11 | Singapore Yacht | 82,843 | 173 | 82,692 | 163 | 81,443 | 83 |
| COI | out35_12 | Singapore Yacht | 52,936 | 280 | 52,759 | 269 | 49,661 | 114 |
| COI | out35_13 | Singapore Yacht | 12,280 | 332 | 12,116 | 326 | 8,773 | 134 |
| COI | out35_14 | Singapore Yacht | 18,345 | 255 | 18,194 | 242 | 15,966 | 98 |
| COI | out35_2 | Singapore Yacht | 60,654 | 508 | 60,458 | 490 | 47,436 | 206 |
| COI | out35_3 | Singapore Yacht | 31,079 | 402 | 30,897 | 386 | 24,116 | 166 |
| COI | out35_4 | Singapore Yacht | 73,031 | 682 | 72,802 | 671 | 48,732 | 267 |
| COI | out35_5 | Singapore Yacht | 37,469 | 285 | 37,314 | 273 | 33,348 | 125 |
| COI | out35_6 | Singapore Yacht | 42,551 | 400 | 42,370 | 390 | 29,668 | 177 |
| COI | out35_7 | Singapore Yacht | 9,533 | 228 | 9,365 | 208 | 7,135 | 89 |
| COI | out35_8 | Singapore Yacht | 99,947 | 743 | 99,693 | 724 | 54,160 | 294 |
| COI | out35_9 | Singapore Yacht | 75,131 | 409 | 74,895 | 396 | 64,934 | 174 |
| COI | outSingapore_105 | Singapore Yacht | 468,602 | 1,034 | 468,383 | 1,028 | 288,494 | 376 |
| COI | outSingapore_106 | Singapore Yacht | 509,933 | 1,003 | 509,706 | 995 | 288,491 | 369 |
| COI | outSingapore_107 | Singapore Yacht | 679,635 | 1,070 | 679,370 | 1,063 | 548,715 | 415 |
| COI | outSingapore_108 | Singapore Yacht | 520,315 | 1,023 | 520,017 | 1,016 | 218,883 | 392 |
| COI | outSingapore_109 | Singapore Yacht | 318,980 | 986 | 318,759 | 977 | 187,520 | 385 |
| COI | outSingapore_110 | Singapore Yacht | 180,927 | 871 | 180,711 | 860 | 98,945 | 337 |
| **COI** | **ALL** | **Singapore Yacht** | **3,410,095** | **2,171** | **3,406,042** | **2,148** | **2,189,453** | **906** |
| COI | out26_07 | Singapore Woodlands | 388,287 | 574 | 388,001 | 570 | 73,399 | 155 |
| COI | out26_08 | Singapore Woodlands | 302,967 | 506 | 302,668 | 504 | 71,337 | 138 |
| COI | out26_09 | Singapore Woodlands | 218,834 | 462 | 218,530 | 459 | 52,613 | 128 |
| COI | out26_10 | Singapore Woodlands | 248,151 | 475 | 247,866 | 471 | 49,732 | 128 |

| Bar-code | Sample ID | Site | Quality Reads | Quality MOTUs | Filtered Reads | Filtered MOTUs | Metazoan Reads | Metazoan MOTUs |
|---|---|---|---|---|---|---|---|---|
| COI | out26_11 | Singapore Woodlands | 376,796 | 580 | 376,495 | 575 | 87,951 | 159 |
| COI | out26_12 | Singapore Woodlands | 300,164 | 591 | 299,879 | 589 | 89,753 | 154 |
| COI | out26_13 | Singapore Woodlands | 413,002 | 619 | 412,591 | 614 | 111,284 | 158 |
| COI | out26_14 | Singapore Woodlands | 320,022 | 571 | 319,589 | 567 | 55,814 | 133 |
| COI | out35_15 | Singapore Woodlands | 351,534 | 514 | 351,224 | 500 | 89,158 | 135 |
| COI | out35_16 | Singapore Woodlands | 177,816 | 411 | 177,515 | 403 | 36,031 | 119 |
| COI | out35_17 | Singapore Woodlands | 62,074 | 270 | 61,792 | 264 | 11,140 | 79 |
| COI | out35_18 | Singapore Woodlands | 98,448 | 331 | 98,160 | 321 | 17,807 | 96 |
| COI | out35_19 | Singapore Woodlands | 74,735 | 238 | 74,454 | 228 | 23,000 | 78 |
| COI | out35_20 | Singapore Woodlands | 54,394 | 259 | 54,109 | 252 | 8,965 | 77 |
| COI | out35_21 | Singapore Woodlands | 30,928 | 155 | 30,661 | 152 | 4,840 | 51 |
| COI | out35_22 | Singapore Woodlands | 85,530 | 262 | 85,248 | 254 | 26,491 | 92 |
| COI | out35_23 | Singapore Woodlands | 382,536 | 483 | 382,245 | 477 | 120,660 | 150 |
| COI | out35_24 | Singapore Woodlands | 154,632 | 334 | 154,351 | 331 | 36,294 | 104 |
| COI | out35_25 | Singapore Woodlands | 39,155 | 229 | 38,875 | 219 | 10,494 | 80 |
| COI | out35_26 | Singapore Woodlands | 57,222 | 279 | 56,944 | 272 | 18,195 | 87 |
| **COI** | **ALL** | **Singapore Woodlands** | **4,137,227** | **1,232** | **4,131,197** | **1,207** | **994,958** | **334** |
| **COI** | **ALL** | **Singapore All Sites** | **7,547,322** | **2,897** | **7,537,239** | **2,867** | **3,184,411** | **1,057** |
| COI | out45_11 | Adelaide Container Channel | 267,952 | 900 | 267,047 | 882 | 217,492 | 387 |
| COI | out45_12 | Adelaide Container Channel | 222,700 | 578 | 221,787 | 552 | 203,253 | 284 |
| COI | out45_13 | Adelaide Container Channel | 216,844 | 737 | 215,992 | 716 | 188,260 | 337 |
| COI | out45_14 | Adelaide Container Channel | 117,833 | 569 | 116,939 | 543 | 102,667 | 266 |
| COI | out45_15 | Adelaide Container Channel | 260,512 | 744 | 259,657 | 715 | 222,905 | 339 |
| COI | out45_16 | Adelaide Container Channel | 106,200 | 747 | 105,364 | 727 | 76,976 | 316 |
| COI | out45_17 | Adelaide Container Channel | 150,874 | 726 | 150,017 | 701 | 120,348 | 306 |
| COI | out45_18 | Adelaide Container Channel | 152,987 | 547 | 152,162 | 518 | 129,800 | 243 |
| COI | out45_19 | Adelaide Container Channel | 152,475 | 629 | 151,593 | 608 | 123,280 | 292 |
| **COI** | **ALL** | **Adelaide Container Channel** | **1,648,377** | **1,761** | **1,640,558** | **1,736** | **1,384,981** | **805** |
| COI | out45_30 | Adelaide Container Dock 1 | 282,149 | 902 | 281,286 | 886 | 233,878 | 387 |
| COI | out45_31 | Adelaide Container Dock 1 | 164,773 | 578 | 163,910 | 551 | 141,913 | 255 |
| COI | out45_32 | Adelaide Container Dock 1 | 183,207 | 556 | 182,346 | 533 | 157,419 | 264 |
| COI | out45_33 | Adelaide Container Dock 1 | 126,774 | 366 | 126,005 | 335 | 122,237 | 163 |
| COI | out45_34 | Adelaide Container Dock 1 | 138,269 | 675 | 137,436 | 644 | 110,838 | 295 |
| COI | out45_35 | Adelaide Container Dock 1 | 134,362 | 518 | 133,582 | 489 | 114,987 | 250 |
| COI | out45_36 | Adelaide Container Dock 1 | 72,223 | 426 | 71,415 | 389 | 56,519 | 195 |
| COI | out45_37 | Adelaide Container Dock 1 | 189,627 | 581 | 188,801 | 555 | 168,697 | 270 |
| COI | out45_38 | Adelaide Container Dock 1 | 141,874 | 609 | 141,014 | 587 | 122,220 | 280 |
| **COI** | **ALL** | **Adelaide Container Dock 1** | **1,433,258** | **1,591** | **1,425,795** | **1,562** | **1,228,708** | **713** |
| COI | out45_29 | Adelaide Container Dock 2 | 146,774 | 569 | 146,024 | 542 | 114,297 | 242 |
| COI | out45_39 | Adelaide Container Dock 2 | 159,086 | 712 | 158,223 | 686 | 133,159 | 319 |
| COI | out45_40 | Adelaide Container Dock 2 | 186,793 | 733 | 185,923 | 714 | 142,557 | 309 |
| COI | out45_41 | Adelaide Container Dock 2 | 131,057 | 552 | 130,221 | 539 | 105,821 | 268 |
| COI | out45_42 | Adelaide Container Dock 2 | 124,635 | 566 | 123,824 | 541 | 96,446 | 255 |
| COI | out45_43 | Adelaide Container Dock 2 | 130,734 | 542 | 129,930 | 518 | 101,400 | 230 |
| COI | out45_44 | Adelaide Container Dock 2 | 143,060 | 617 | 142,193 | 596 | 118,368 | 273 |
| COI | out45_45 | Adelaide Container Dock 2 | 158,338 | 688 | 157,481 | 664 | 126,677 | 297 |

| Bar-code | Sample ID | Site | Quality Reads | Quality MOTUs | Filtered Reads | Filtered MOTUs | Metazoan Reads | Metazoan MOTUs |
|---|---|---|---|---|---|---|---|---|
| COI | out45_46 | Adelaide Container Dock 2 | 141,661 | 629 | 140,774 | 602 | 110,466 | 293 |
| COI | out45_47 | Adelaide Container Dock 2 | 159,797 | 667 | 159,003 | 645 | 115,019 | 290 |
| **COI** | **ALL** | **Adelaide Container Dock 2** | **1,481,935** | **1,651** | **1,473,596** | **1,627** | **1,164,210** | **736** |
| COI | out44_10 | Fuel Channel | 314,950 | 758 | 314,078 | 736 | 254,067 | 335 |
| COI | out44_11 | Fuel Channel | 263,283 | 574 | 262,435 | 552 | 230,811 | 273 |
| COI | out44_12 | Fuel Channel | 172,723 | 466 | 171,886 | 448 | 151,807 | 221 |
| COI | out44_13 | Fuel Channel | 219,641 | 605 | 218,791 | 574 | 192,567 | 267 |
| COI | out44_14 | Fuel Channel | 156,968 | 601 | 156,110 | 583 | 127,248 | 274 |
| COI | out44_16 | Fuel Channel | 173,327 | 633 | 172,560 | 615 | 149,931 | 287 |
| COI | out44_17 | Fuel Channel | 178,048 | 678 | 177,277 | 661 | 142,095 | 306 |
| COI | out44_18 | Fuel Channel | 248,580 | 729 | 247,799 | 714 | 205,329 | 329 |
| COI | out44_19 | Fuel Channel | 345,681 | 770 | 344,807 | 757 | 291,024 | 352 |
| **COI** | **ALL** | **Fuel Channel** | **2,073,201** | **1,502** | **2,065,743** | **1,479** | **1,744,879** | **659** |
| COI | out44_1 | Adelaide Fuel Dock | 123,614 | 600 | 122,840 | 580 | 100,172 | 270 |
| COI | out44_2 | Adelaide Fuel Dock | 147,682 | 744 | 146,789 | 722 | 111,387 | 334 |
| COI | out44_3 | Adelaide Fuel Dock | 224,130 | 761 | 223,295 | 744 | 170,038 | 332 |
| COI | out44_4 | Adelaide Fuel Dock | 249,081 | 653 | 248,213 | 626 | 218,584 | 290 |
| COI | out44_5 | Adelaide Fuel Dock | 358,233 | 922 | 357,329 | 903 | 276,160 | 400 |
| COI | out44_6 | Adelaide Fuel Dock | 212,170 | 797 | 211,303 | 782 | 171,561 | 366 |
| COI | out44_7 | Adelaide Fuel Dock | 261,488 | 900 | 260,580 | 880 | 217,419 | 389 |
| COI | out44_8 | Adelaide Fuel Dock | 162,150 | 652 | 161,278 | 631 | 141,807 | 288 |
| COI | out44_9 | Adelaide Fuel Dock | 209,425 | 656 | 208,507 | 635 | 183,457 | 315 |
| COI | out45_28 | Adelaide Fuel Dock | 156,299 | 636 | 155,415 | 605 | 125,696 | 286 |
| **COI** | **ALL** | **Adelaide Fuel Dock** | **2,104,272** | **1,821** | **2,095,549** | **1,797** | **1,716,281** | **811** |
| COI | out45_1 | Adelaide Marina Channel | 187,266 | 790 | 186,351 | 768 | 150,588 | 361 |
| COI | out45_2 | Adelaide Marina Channel | 188,093 | 722 | 187,250 | 706 | 153,276 | 305 |
| COI | out45_27 | Adelaide Marina Channel | 164,537 | 649 | 163,727 | 625 | 136,365 | 281 |
| COI | out45_3 | Adelaide Marina Channel | 171,480 | 860 | 170,663 | 840 | 135,541 | 377 |
| COI | out45_4 | Adelaide Marina Channel | 228,476 | 871 | 227,648 | 847 | 182,217 | 387 |
| COI | out45_5 | Adelaide Marina Channel | 256,815 | 772 | 255,947 | 747 | 212,373 | 344 |
| COI | out45_6 | Adelaide Marina Channel | 187,529 | 754 | 186,669 | 734 | 159,465 | 335 |
| COI | out45_7 | Adelaide Marina Channel | 230,229 | 812 | 229,380 | 791 | 194,245 | 352 |
| COI | out45_8 | Adelaide Marina Channel | 172,628 | 732 | 171,840 | 711 | 136,813 | 339 |
| COI | out45_9 | Adelaide Marina Channel | 239,756 | 775 | 238,961 | 760 | 199,106 | 328 |
| **COI** | **ALL** | **Adelaide Marina Channel** | **2,026,809** | **1,985** | **2,018,436** | **1,958** | **1,659,989** | **882** |
| COI | out44_39 | Adelaide Marina Dock | 121,188 | 523 | 120,295 | 505 | 107,569 | 227 |
| COI | out44_40 | Adelaide Marina Dock | 49,079 | 336 | 48,320 | 310 | 40,591 | 152 |
| COI | out44_41 | Adelaide Marina Dock | 191,553 | 591 | 190,631 | 576 | 169,749 | 266 |
| COI | out44_42 | Adelaide Marina Dock | 229,986 | 811 | 229,020 | 796 | 190,478 | 352 |
| COI | out44_43 | Adelaide Marina Dock | 133,192 | 665 | 132,207 | 656 | 103,057 | 296 |
| COI | out44_45 | Adelaide Marina Dock | 519,126 | 923 | 518,169 | 910 | 419,323 | 393 |
| COI | out44_46 | Adelaide Marina Dock | 282,925 | 813 | 282,075 | 798 | 226,087 | 340 |
| COI | out44_47 | Adelaide Marina Dock | 309,827 | 784 | 308,983 | 769 | 281,467 | 359 |
| COI | out44_48 | Adelaide Marina Dock | 193,321 | 567 | 192,513 | 546 | 158,612 | 271 |
| **COI** | **ALL** | **Adelaide Marina Dock** | **2,030,197** | **1,678** | **2,022,213** | **1,664** | **1,696,933** | **716** |
| **COI** | **ALL** | **Adelaide All Sites** | **12,798,049** | **3,491** | **12,741,890** | **3,467** | **10,595,981** | **1,579** |

**Supplementary Figure S1.** Rarefied metMOTU accumulation curves by read count (# Metazoan sequences) for each sample in the 18S dataset. Samples from each of 11 sites (a-k) are labelled corresponding to their ID in the dataset.

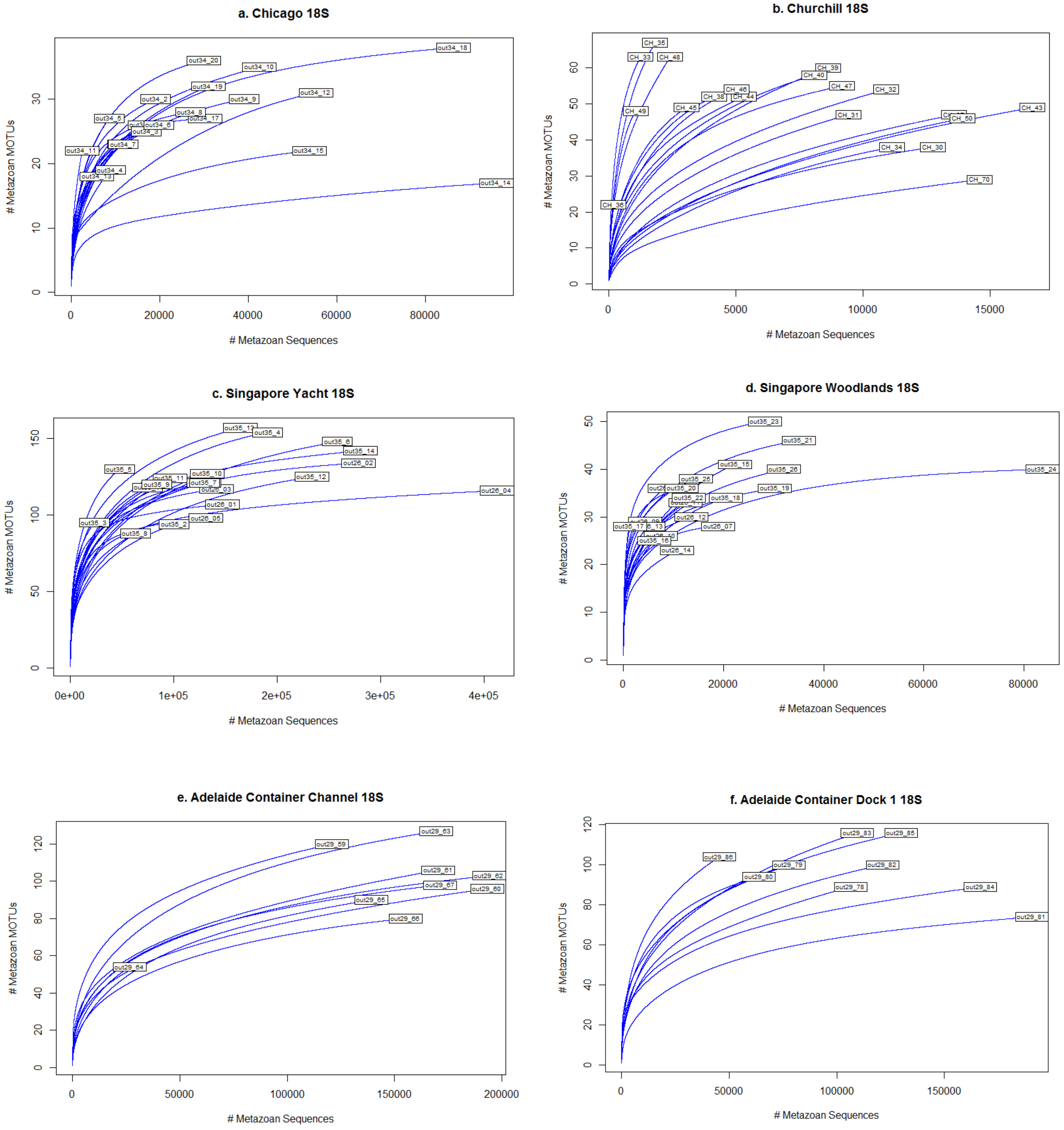

**g. Adelaide Container Dock 2 18S**

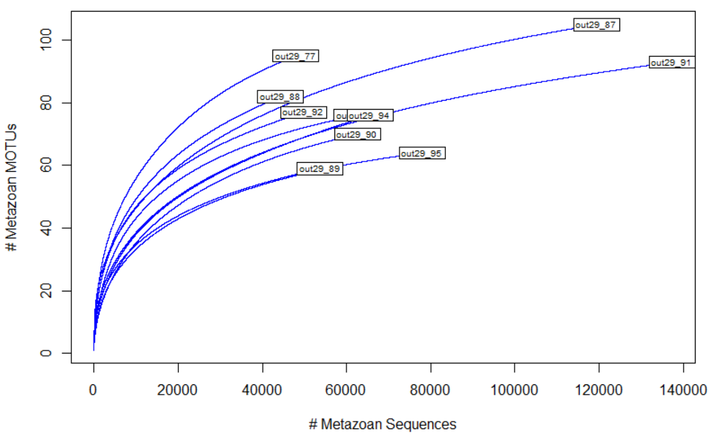


**h. Adelaide Fuel Channel 18S**

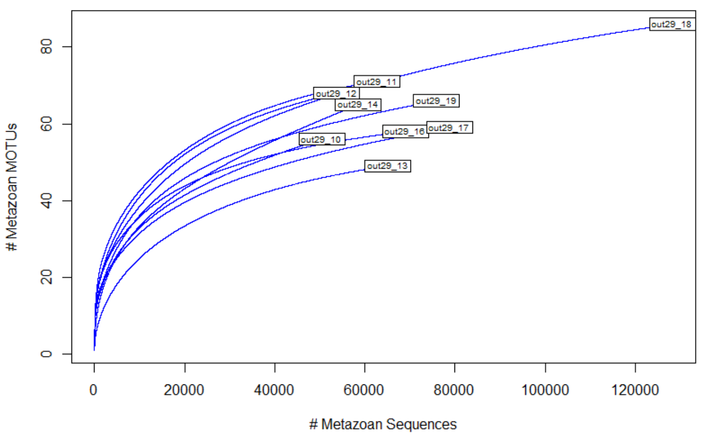


**i. Adelaide Fuel Dock 18S**

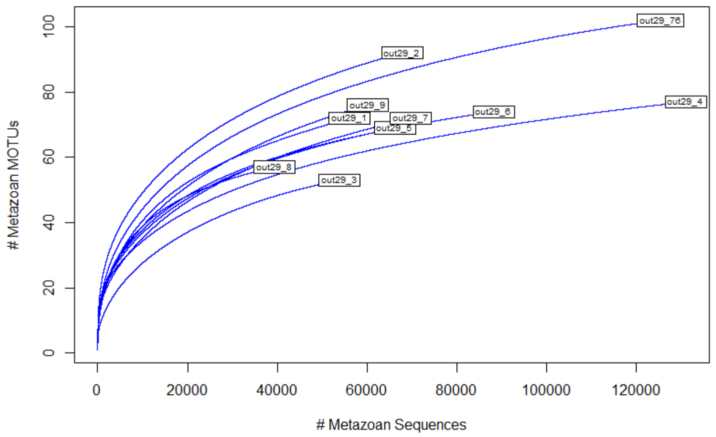


**j. Adelaide Marina Channel 18S**

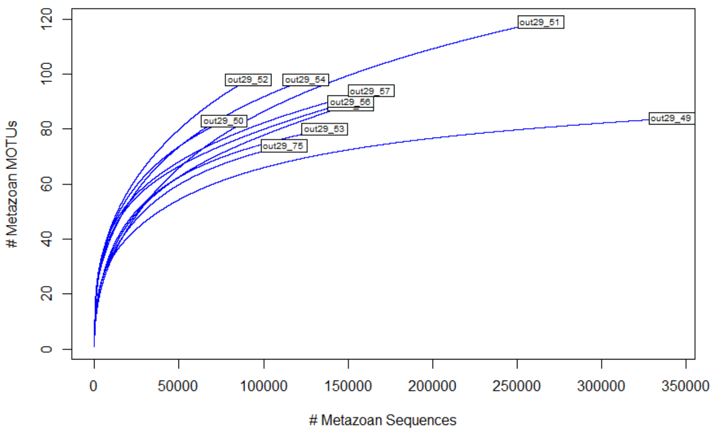


**k. Adelaide Marina Dock 18S**

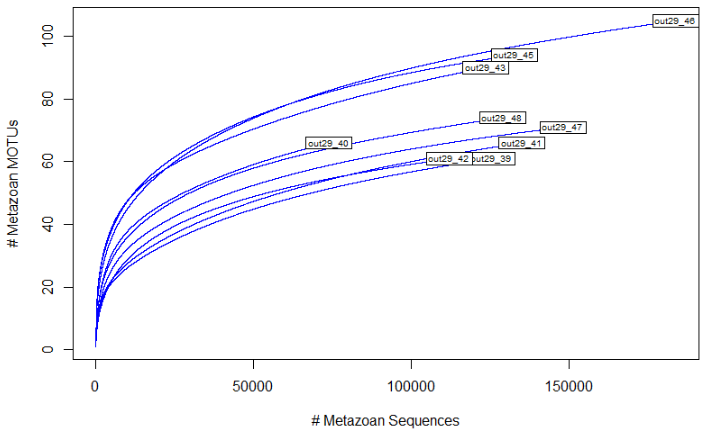

**Supplementary Table S2.** Number of reads, observed shared species, estimated shared species (Chao1 estimator) and the observed/Chao1 ratio for each sample in the 18S dataset.

| Site | Sample | # Reads | # Observed metMOTUs | Chao1 Estimate | Chao 1 Std. Error | Observed/Chao1 |
|---|---|---|---|---|---|---|
| Churchill | CH_30 | 12,756 | 38 | 47.8 | 7.2 | 0.80 |
| Churchill | CH_31 | 9,417 | 47 | 68.4 | 13.1 | 0.69 |
| Churchill | CH_32 | 10,916 | 54 | 80.3 | 15.5 | 0.67 |
| Churchill | CH_33 | 1,240 | 63 | 73.0 | 6.7 | 0.86 |
| Churchill | CH_34 | 11,135 | 38 | 47.5 | 6.6 | 0.80 |
| Churchill | CH_35 | 1,813 | 67 | 109.2 | 24.7 | 0.61 |
| Churchill | CH_36 | 185 | 22 | 40.3 | 15.0 | 0.55 |
| Churchill | CH_37 | 13,579 | 47 | 68.4 | 13.1 | 0.69 |
| Churchill | CH_38 | 4,137 | 52 | 58.0 | 4.5 | 0.90 |
| Churchill | CH_39 | 8,622 | 60 | 85.3 | 14.1 | 0.70 |
| Churchill | CH_40 | 8,078 | 58 | 70.4 | 7.9 | 0.82 |
| Churchill | CH_43 | 16,670 | 49 | 70.0 | 12.2 | 0.70 |
| Churchill | CH_44 | 5,310 | 52 | 74.7 | 14.9 | 0.70 |
| Churchill | CH_45 | 3,054 | 49 | 58.1 | 6.5 | 0.84 |
| Churchill | CH_46 | 5,024 | 54 | 61.0 | 5.1 | 0.89 |
| Churchill | CH_47 | 9,165 | 55 | 66.4 | 8.1 | 0.83 |
| Churchill | CH_48 | 2,406 | 63 | 133.2 | 40.2 | 0.47 |
| Churchill | CH_49 | 1,049 | 48 | 52.7 | 3.8 | 0.91 |
| Churchill | CH_50 | 13,922 | 46 | 67.1 | 12.6 | 0.69 |
| Churchill | CH_70 | 14,585 | 29 | 48.5 | 14.7 | 0.60 |
| **Churchill ALL** | | **7,653** | **49.6** | **69.0** | | **0.74** |
| Chicago | out34_1 | 11,543 | 23 | 25.1 | 2.5 | 0.91 |
| Chicago | out34_10 | 42,471 | 35 | 44.3 | 8.8 | 0.79 |
| Chicago | out34_11 | 2,406 | 22 | 31.0 | 8.0 | 0.71 |
| Chicago | out34_12 | 55,181 | 31 | 33.5 | 2.6 | 0.92 |
| Chicago | out34_13 | 5,726 | 18 | 18.3 | 0.7 | 0.99 |
| Chicago | out34_14 | 95,989 | 17 | 18.5 | 2.2 | 0.92 |
| Chicago | out34_15 | 53,719 | 22 | 23.2 | 1.8 | 0.95 |
| Chicago | out34_16 | 16,573 | 26 | 33.0 | 7.1 | 0.79 |
| Chicago | out34_17 | 30,128 | 27 | 27.0 | 0.1 | 1.00 |
| Chicago | out34_18 | 86,225 | 38 | 40.0 | 2.6 | 0.95 |
| Chicago | out34_19 | 30,899 | 32 | 39.2 | 6.4 | 0.82 |
| Chicago | out34_2 | 18,936 | 30 | 33.8 | 4.2 | 0.89 |
| Chicago | out34_20 | 29,947 | 36 | 37.7 | 2.2 | 0.96 |
| Chicago | out34_3 | 16,827 | 25 | 25.5 | 1.0 | 0.98 |
| Chicago | out34_4 | 8,827 | 19 | 24.3 | 5.4 | 0.78 |
| Chicago | out34_5 | 8,526 | 27 | 34.0 | 6.6 | 0.79 |
| Chicago | out34_6 | 19,682 | 26 | 28.5 | 3.2 | 0.91 |
| Chicago | out34_7 | 11,696 | 23 | 25.5 | 3.2 | 0.90 |
| Chicago | out34_8 | 26,836 | 28 | 28.6 | 1.2 | 0.98 |
| Chicago | out34_9 | 38,961 | 30 | 31.4 | 1.9 | 0.95 |
| **Chicago ALL** | | **30,555** | **26.8** | **30.1** | | **0.89** |
| Singapore Yacht | out26_01 | 146,731 | 107 | 111.5 | 4.1 | 0.96 |
| Singapore Yacht | out26_02 | 278,600 | 134 | 139.1 | 4.0 | 0.96 |
| Singapore Yacht | out26_03 | 141,200 | 117 | 123.5 | 4.9 | 0.95 |
| Singapore Yacht | out26_04 | 412,363 | 116 | 129.0 | 9.3 | 0.90 |
| Singapore Yacht | out26_05 | 131,082 | 98 | 101.1 | 3.1 | 0.97 |
| Singapore Yacht | out26_06 | 129,051 | 121 | 130.5 | 6.6 | 0.93 |
| Singapore Yacht | out35_1 | 74,476 | 118 | 130.0 | 6.7 | 0.91 |
| Singapore Yacht | out35_10 | 132,189 | 127 | 143.2 | 8.0 | 0.89 |
| Singapore Yacht | out35_11 | 96,007 | 124 | 143.1 | 9.9 | 0.87 |
| Singapore Yacht | out35_12 | 233,286 | 125 | 142.6 | 9.3 | 0.88 |

| Site | Sample | # Reads | # Observed metMOTUs | Chao1 Estimate | Chao 1 Std. Error | Observed/Chao1 |
|---|---|---|---|---|---|---|
| Singapore Yacht | out35_13 | 164,178 | 157 | 178.4 | 10.5 | 0.88 |
| Singapore Yacht | out35_14 | 280,014 | 142 | 159.0 | 10.7 | 0.89 |
| Singapore Yacht | out35_2 | 100,291 | 94 | 124.0 | 16.2 | 0.76 |
| Singapore Yacht | out35_3 | 23,088 | 95 | 109.6 | 8.6 | 0.87 |
| Singapore Yacht | out35_4 | 190,589 | 154 | 163.7 | 5.3 | 0.94 |
| Singapore Yacht | out35_5 | 47,513 | 130 | 146.3 | 8.4 | 0.89 |
| Singapore Yacht | out35_6 | 258,138 | 148 | 168.2 | 9.6 | 0.88 |
| Singapore Yacht | out35_7 | 129,575 | 121 | 140.7 | 10.6 | 0.86 |
| Singapore Yacht | out35_8 | 62,644 | 88 | 113.3 | 14.1 | 0.78 |
| Singapore Yacht | out35_9 | 83,720 | 120 | 159.2 | 17.2 | 0.75 |
| **Singapore Yacht ALL** | | **155,737** | **121.8** | **137.8** | | **0.89** |
| Singapore Woodlands | out26_07 | 18,951 | 28 | 28.0 | 0.1 | 1.00 |
| Singapore Woodlands | out26_08 | 8,172 | 36 | 41.3 | 5.4 | 0.87 |
| Singapore Woodlands | out26_09 | 4,332 | 29 | 31.5 | 3.2 | 0.92 |
| Singapore Woodlands | out26_10 | 7,495 | 26 | 29.8 | 4.2 | 0.87 |
| Singapore Woodlands | out26_11 | 12,406 | 33 | 42.0 | 8.0 | 0.79 |
| Singapore Woodlands | out26_12 | 13,567 | 30 | 35.0 | 6.0 | 0.86 |
| Singapore Woodlands | out26_13 | 4,985 | 28 | 30.5 | 3.2 | 0.92 |
| Singapore Woodlands | out26_14 | 10,676 | 23 | 28.0 | 5.5 | 0.82 |
| Singapore Woodlands | out35_15 | 22,321 | 41 | 46.0 | 4.3 | 0.89 |
| Singapore Woodlands | out35_16 | 6,169 | 25 | 28.3 | 4.1 | 0.88 |
| Singapore Woodlands | out35_17 | 1,296 | 28 | 30.5 | 2.9 | 0.92 |
| Singapore Woodlands | out35_18 | 20,507 | 34 | 37.0 | 3.2 | 0.92 |
| Singapore Woodlands | out35_19 | 30,252 | 36 | 38.5 | 3.2 | 0.94 |
| Singapore Woodlands | out35_20 | 11,632 | 36 | 36.8 | 1.4 | 0.98 |
| Singapore Woodlands | out35_21 | 35,012 | 46 | 47.9 | 2.3 | 0.96 |
| Singapore Woodlands | out35_22 | 13,103 | 34 | 45.3 | 9.5 | 0.75 |
| Singapore Woodlands | out35_23 | 28,206 | 50 | 51.7 | 2.2 | 0.97 |
| Singapore Woodlands | out35_24 | 83,716 | 40 | 40.6 | 1.2 | 0.99 |
| Singapore Woodlands | out35_25 | 14,502 | 38 | 41.0 | 3.4 | 0.93 |
| Singapore Woodlands | out35_26 | 31,997 | 40 | 47.0 | 6.6 | 0.85 |
| **Singapore Woodlands ALL** | | **18,965** | **34.1** | **37.8** | | **0.90** |
| Adelaide Container Channel | out29_59 | 120,748 | 120 | 147.2 | 13.1 | 0.82 |
| Adelaide Container Channel | out29_60 | 193,117 | 96 | 138.3 | 20.7 | 0.69 |
| Adelaide Container Channel | out29_61 | 170,309 | 106 | 160.1 | 25.3 | 0.66 |
| Adelaide Container Channel | out29_62 | 194,122 | 103 | 118.8 | 8.7 | 0.87 |
| Adelaide Container Channel | out29_63 | 169,492 | 127 | 152.8 | 12.2 | 0.83 |
| Adelaide Container Channel | out29_64 | 26,464 | 54 | 73.0 | 11.3 | 0.74 |
| Adelaide Container Channel | out29_65 | 139,165 | 90 | 113.2 | 12.0 | 0.79 |
| Adelaide Container Channel | out29_66 | 155,178 | 80 | 89.0 | 5.7 | 0.90 |
| Adelaide Container Channel | out29_67 | 171,224 | 98 | 113.4 | 8.7 | 0.86 |
| **Adelaide Container Channel ALL** | | **148,869** | **97.1** | **122.9** | | **0.80** |
| Adelaide Container Dock 1 | out29_78 | 106,377 | 89 | 127.8 | 18.8 | 0.70 |
| Adelaide Container Dock 1 | out29_79 | 77,647 | 100 | 139.5 | 19.6 | 0.72 |
| Adelaide Container Dock 1 | out29_80 | 63,856 | 94 | 105.4 | 7.0 | 0.89 |
| Adelaide Container Dock 1 | out29_81 | 190,675 | 74 | 101.2 | 18.2 | 0.73 |
| Adelaide Container Dock 1 | out29_82 | 121,166 | 100 | 125.2 | 12.6 | 0.80 |
| Adelaide Container Dock 1 | out29_83 | 109,594 | 116 | 162.3 | 19.9 | 0.71 |
| Adelaide Container Dock 1 | out29_84 | 166,747 | 89 | 128.4 | 22.4 | 0.69 |
| Adelaide Container Dock 1 | out29_85 | 129,799 | 116 | 147.0 | 14.8 | 0.79 |
| Adelaide Container Dock 1 | out29_86 | 45,389 | 104 | 139.4 | 16.8 | 0.75 |
| **Adelaide Container Dock 1 ALL** | | **112,361** | **98.0** | **130.7** | | **0.75** |
| Adelaide Container Dock 2 | out29_77 | 47,951 | 95 | 115.0 | 10.6 | 0.83 |
| Adelaide Container Dock 2 | out29_87 | 119,395 | 105 | 124.1 | 9.9 | 0.85 |
| Adelaide Container Dock 2 | out29_88 | 44,267 | 82 | 112.0 | 16.2 | 0.73 |

| Site | Sample | # Reads | # Observed metMOTUs | Chao1 Estimate | Chao 1 Std. Error | Observed/Chao1 |
|---|---|---|---|---|---|---|
| Adelaide Container Dock 2 | out29_89 | 53,748 | 59 | 72.3 | 8.8 | 0.82 |
| Adelaide Container Dock 2 | out29_90 | 62,571 | 70 | 83.2 | 8.0 | 0.84 |
| Adelaide Container Dock 2 | out29_91 | 137,374 | 93 | 120.3 | 14.6 | 0.77 |
| Adelaide Container Dock 2 | out29_92 | 49,728 | 77 | 110.0 | 19.3 | 0.70 |
| Adelaide Container Dock 2 | out29_93 | 62,441 | 76 | 88.4 | 8.0 | 0.86 |
| Adelaide Container Dock 2 | out29_94 | 65,559 | 76 | 115.0 | 20.6 | 0.66 |
| Adelaide Container Dock 2 | out29_95 | 77,985 | 64 | 70.0 | 4.5 | 0.91 |
| **Adelaide Container Dock 2 ALL** | | **72,102** | **79.7** | **101.0** | | **0.80** |
| Adelaide Fuel Channel | out29_10 | 50,408 | 56 | 84.5 | 17.9 | 0.66 |
| Adelaide Fuel Channel | out29_11 | 62,767 | 71 | 74.7 | 3.1 | 0.95 |
| Adelaide Fuel Channel | out29_12 | 53,742 | 68 | 78.5 | 7.2 | 0.87 |
| Adelaide Fuel Channel | out29_13 | 65,074 | 49 | 53.6 | 3.8 | 0.91 |
| Adelaide Fuel Channel | out29_14 | 58,547 | 65 | 191.0 | 77.4 | 0.34 |
| Adelaide Fuel Channel | out29_16 | 69,046 | 58 | 63.6 | 4.8 | 0.91 |
| Adelaide Fuel Channel | out29_17 | 78,816 | 59 | 74.0 | 10.0 | 0.80 |
| Adelaide Fuel Channel | out29_18 | 128,289 | 86 | 107.0 | 11.9 | 0.80 |
| Adelaide Fuel Channel | out29_19 | 75,926 | 66 | 88.7 | 14.9 | 0.74 |
| **Adelaide Fuel Channel ALL** | | **71,402** | **64.2** | **90.6** | | **0.78** |
| Adelaide Fuel Dock | out29_1 | 56,093 | 72 | 110.5 | 22.9 | 0.65 |
| Adelaide Fuel Dock | out29_2 | 67,786 | 92 | 109.6 | 9.3 | 0.84 |
| Adelaide Fuel Dock | out29_3 | 53,840 | 53 | 61.8 | 6.0 | 0.86 |
| Adelaide Fuel Dock | out29_4 | 131,009 | 77 | 96.0 | 11.3 | 0.80 |
| Adelaide Fuel Dock | out29_5 | 66,238 | 69 | 86.1 | 10.4 | 0.80 |
| Adelaide Fuel Dock | out29_6 | 88,142 | 74 | 89.3 | 9.6 | 0.83 |
| Adelaide Fuel Dock | out29_7 | 69,713 | 72 | 100.1 | 15.8 | 0.72 |
| Adelaide Fuel Dock | out29_76 | 125,316 | 102 | 113.5 | 6.4 | 0.90 |
| Adelaide Fuel Dock | out29_8 | 39,353 | 57 | 63.6 | 5.1 | 0.90 |
| Adelaide Fuel Dock | out29_9 | 60,184 | 76 | 91.8 | 8.7 | 0.83 |
| **Adelaide Fuel Dock ALL** | | **75,767** | **74.4** | **92.2** | | **0.81** |
| Adelaide Marina Channel | out29_49 | 341,688 | 84 | 102.2 | 13.2 | 0.82 |
| Adelaide Marina Channel | out29_50 | 76,710 | 83 | 104.0 | 11.9 | 0.80 |
| Adelaide Marina Channel | out29_51 | 264,013 | 119 | 161.0 | 18.8 | 0.74 |
| Adelaide Marina Channel | out29_52 | 91,090 | 98 | 123.8 | 12.2 | 0.79 |
| Adelaide Marina Channel | out29_53 | 136,267 | 80 | 105.5 | 16.4 | 0.76 |
| Adelaide Marina Channel | out29_54 | 125,142 | 98 | 125.0 | 13.5 | 0.78 |
| Adelaide Marina Channel | out29_55 | 151,492 | 89 | 151.1 | 32.7 | 0.59 |
| Adelaide Marina Channel | out29_56 | 151,704 | 90 | 122.5 | 17.2 | 0.73 |
| Adelaide Marina Channel | out29_57 | 163,569 | 94 | 140.4 | 25.6 | 0.67 |
| Adelaide Marina Channel | out29_75 | 112,064 | 74 | 89.1 | 9.7 | 0.83 |
| **Adelaide Marina Channel ALL** | | **161,374** | **90.9** | **122.5** | | **0.75** |
| Adelaide Marina Dock | out29_39 | 125,488 | 61 | 82.4 | 13.1 | 0.74 |
| Adelaide Marina Dock | out29_40 | 73,924 | 66 | 81.3 | 9.6 | 0.81 |
| Adelaide Marina Dock | out29_41 | 135,102 | 66 | 85.0 | 11.3 | 0.78 |
| Adelaide Marina Dock | out29_42 | 112,110 | 61 | 76.0 | 10.0 | 0.80 |
| Adelaide Marina Dock | out29_43 | 123,599 | 90 | 115.1 | 13.7 | 0.78 |
| Adelaide Marina Dock | out29_45 | 132,650 | 94 | 107.2 | 8.0 | 0.88 |
| Adelaide Marina Dock | out29_46 | 183,898 | 105 | 134.5 | 15.5 | 0.78 |
| Adelaide Marina Dock | out29_47 | 148,147 | 71 | 88.0 | 10.7 | 0.81 |
| Adelaide Marina Dock | out29_48 | 128,867 | 74 | 98.4 | 15.2 | 0.75 |
| **Adelaide Marina Dock ALL** | | **129,309** | **76.4** | **96.4** | | **0.79** |

**Supplementary Figure S2.** Rarefied metMOTU accumulation by read count (# metazoan sequences) curves for each sample in the COI dataset. Samples from each of 11 sites (a – k) are labelled corresponding to their ID in the dataset.

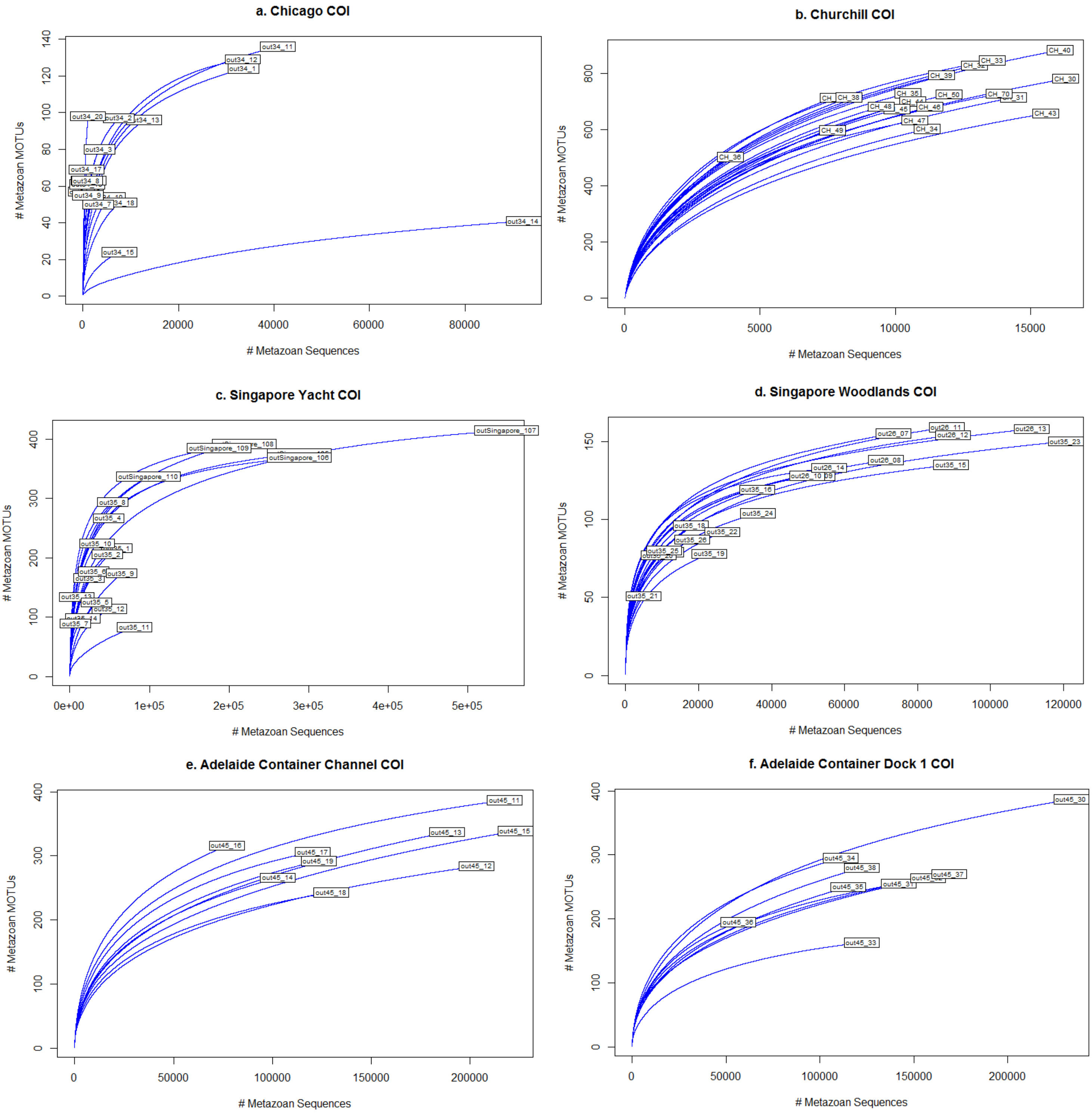

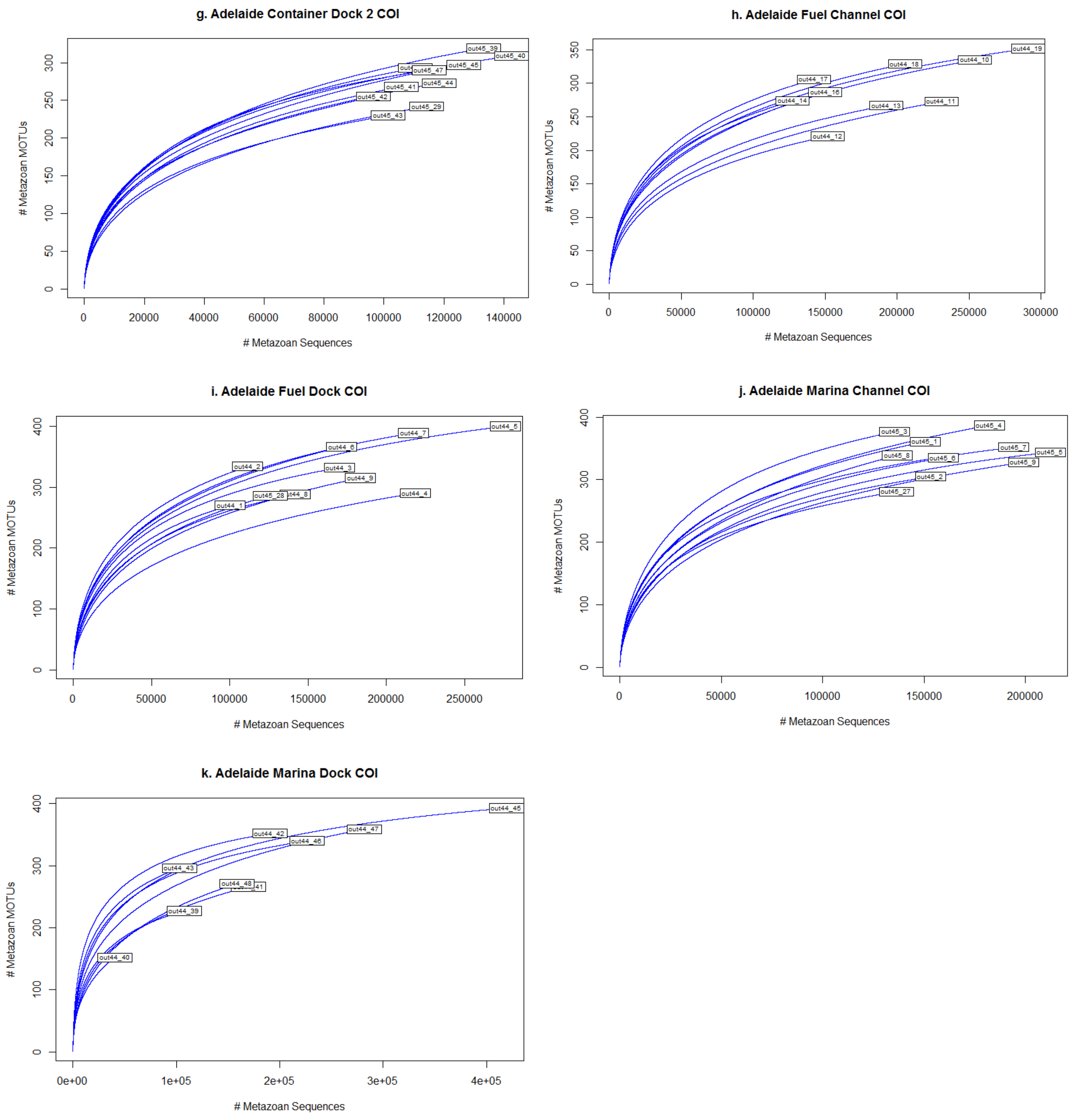
g. Adelaide Container Dock 2 COI
# Metazoan MOTUs
0
50
100
150
200
250
300
# Metazoan Sequences
0
20000
40000
60000
80000
100000
120000
140000
out45_39
out45_40
out45_45
out45_47
out45_44
out45_41
out45_42
out45_29
out45_43
h. Adelaide Fuel Channel COI
# Metazoan MOTUs
0
50
100
150
200
250
300
350
# Metazoan Sequences
0
50000
100000
150000
200000
250000
300000
out44_19
out44_10
out44_18
out44_17
out44_16
out44_14
out44_13
out44_11
out44_12
i. Adelaide Fuel Dock COI
# Metazoan MOTUs
0
100
200
300
400
# Metazoan Sequences
0
50000
100000
150000
200000
250000
out44_5
out44_7
out44_6
out44_2
out44_3
out44_9
out45_28
out44_1
out44_4
j. Adelaide Marina Channel COI
# Metazoan MOTUs
0
100
200
300
400
# Metazoan Sequences
0
50000
100000
150000
200000
out45_3
out45_4
out45_1
out45_8
out45_6
out45_7
out45_5
out45_9
out45_2
out45_27
k. Adelaide Marina Dock COI
# Metazoan MOTUs
0
100
200
300
400
# Metazoan Sequences
0e+00
1e+05
2e+05
3e+05
4e+05
out44_45
out44_42
out44_46
out44_47
out44_43
out44_48
out44_39
out44_40

**Supplementary Table S3.** Number of reads, observed metMOTUs, estimated metMOTUs (Chao1 estimator) and the observed richness/Chao1 estimate ratio in the COI dataset.

| Site | Sample ID | Number of Reads | Observed metMOTUs | Chao1 Estimate | Chao 1 Std. Error | Observed/Chao1 |
|---|---|---|---|---|---|---|
| Churchill | CH_30 | 16,314 | 782 | 1084.6 | 48.1 | 0.72 |
| Churchill | CH_31 | 14,378 | 715 | 1005.7 | 47.2 | 0.71 |
| Churchill | CH_32 | 12,935 | 829 | 1077.0 | 39.3 | 0.77 |
| Churchill | CH_33 | 13,578 | 847 | 1029.4 | 31.1 | 0.82 |
| Churchill | CH_34 | 11,179 | 603 | 846.0 | 40.9 | 0.71 |
| Churchill | CH_35 | 10,466 | 730 | 944.4 | 35.7 | 0.77 |
| Churchill | CH_36 | 3,902 | 503 | 840.9 | 60.9 | 0.60 |
| Churchill | CH_37 | 7,695 | 714 | 974.0 | 41.2 | 0.73 |
| Churchill | CH_38 | 8,290 | 715 | 1010.2 | 46.1 | 0.71 |
| Churchill | CH_39 | 11,718 | 794 | 1042.2 | 39.2 | 0.76 |
| Churchill | CH_40 | 16,105 | 884 | 1167.2 | 43.8 | 0.76 |
| Churchill | CH_43 | 15,562 | 658 | 921.4 | 43.9 | 0.71 |
| Churchill | CH_44 | 10,644 | 701 | 1002.1 | 46.5 | 0.70 |
| Churchill | CH_45 | 10,066 | 674 | 977.2 | 49.5 | 0.69 |
| Churchill | CH_46 | 11,275 | 682 | 990.0 | 49.3 | 0.69 |
| Churchill | CH_47 | 10,715 | 633 | 820.9 | 34.9 | 0.77 |
| Churchill | CH_48 | 9,483 | 683 | 962.3 | 45.0 | 0.71 |
| Churchill | CH_49 | 7,670 | 598 | 869.9 | 47.4 | 0.69 |
| Churchill | CH_50 | 11,985 | 726 | 951.9 | 37.4 | 0.76 |
| Churchill | CH_70 | 13,842 | 728 | 955.0 | 37.9 | 0.76 |
| **Churchill ALL** | | **11,390** | **710.0** | **973.6** | | **0.73** |
| Chicago | out34_1 | 33,529 | 124 | 151.3 | 14.6 | 0.82 |
| Chicago | out34_10 | 5,202 | 54 | 79.7 | 14.7 | 0.68 |
| Chicago | out34_11 | 40,777 | 136 | 154.1 | 9.3 | 0.88 |
| Chicago | out34_12 | 33,445 | 129 | 134.0 | 3.6 | 0.96 |
| Chicago | out34_13 | 12,851 | 96 | 117.2 | 11.5 | 0.82 |
| Chicago | out34_14 | 92,281 | 41 | 60.1 | 12.0 | 0.68 |
| Chicago | out34_15 | 7,597 | 24 | 31.5 | 6.3 | 0.76 |
| Chicago | out34_16 | 530 | 57 | 68.3 | 7.3 | 0.83 |
| Chicago | out34_17 | 773 | 69 | 100.2 | 15.5 | 0.69 |
| Chicago | out34_18 | 7,640 | 51 | 61.5 | 6.8 | 0.83 |
| Chicago | out34_19 | 896 | 61 | 73.8 | 8.0 | 0.83 |
| Chicago | out34_2 | 7,507 | 97 | 112.8 | 9.4 | 0.86 |
| Chicago | out34_20 | 1,074 | 98 | 122.5 | 11.6 | 0.80 |
| Chicago | out34_3 | 3,426 | 80 | 95.3 | 9.6 | 0.84 |
| Chicago | out34_4 | 780 | 63 | 95.5 | 17.2 | 0.66 |
| Chicago | out34_5 | 749 | 63 | 82.1 | 11.0 | 0.77 |
| Chicago | out34_6 | 1,592 | 63 | 90.3 | 14.6 | 0.70 |
| Chicago | out34_7 | 3,190 | 50 | 75.5 | 16.4 | 0.66 |
| Chicago | out34_8 | 878 | 63 | 102.4 | 22.4 | 0.62 |
| Chicago | out34_9 | 1,069 | 55 | 68.2 | 8.0 | 0.81 |
| **Chicago ALL** | | **12,789** | **73.7** | **93.8** | | **0.77** |
| Singapore Yacht | out35_1 | 59,156 | 216 | 261.0 | 15.4 | 0.83 |
| Singapore Yacht | out35_10 | 33,877 | 224 | 265.2 | 13.9 | 0.84 |
| Singapore Yacht | out35_11 | 81,443 | 83 | 138.7 | 24.1 | 0.60 |
| Singapore Yacht | out35_12 | 49,661 | 114 | 157.3 | 18.3 | 0.72 |
| Singapore Yacht | out35_13 | 8,773 | 134 | 150.0 | 7.7 | 0.89 |
| Singapore Yacht | out35_14 | 15,966 | 98 | 119.0 | 10.5 | 0.82 |
| Singapore Yacht | out35_2 | 47,436 | 206 | 241.4 | 13.0 | 0.85 |
| Singapore Yacht | out35_3 | 24,116 | 166 | 210.0 | 16.2 | 0.79 |
| Singapore Yacht | out35_4 | 48,732 | 267 | 313.2 | 15.0 | 0.85 |
| Singapore Yacht | out35_5 | 33,348 | 125 | 178.1 | 19.8 | 0.70 |

| Site | Sample ID | Number of Reads | Observed metMOTUs | Chao1 Estimate | Chao 1 Std. Error | Observed/Chao1 |
|---|---|---|---|---|---|---|
| Singapore Yacht | out35_6 | 29,668 | 177 | 251.0 | 24.3 | 0.71 |
| Singapore Yacht | out35_7 | 7,135 | 89 | 120.1 | 14.3 | 0.74 |
| Singapore Yacht | out35_8 | 54,160 | 294 | 369.1 | 25.5 | 0.80 |
| Singapore Yacht | out35_9 | 64,934 | 174 | 307.0 | 42.8 | 0.57 |
| Singapore Yacht | outSingapore_105 | 288,494 | 376 | 404.7 | 11.6 | 0.93 |
| Singapore Yacht | outSingapore_106 | 288,491 | 369 | 387.1 | 8.2 | 0.95 |
| Singapore Yacht | outSingapore_107 | 548,715 | 415 | 437.0 | 8.3 | 0.95 |
| Singapore Yacht | outSingapore_108 | 218,883 | 392 | 417.1 | 10.7 | 0.94 |
| Singapore Yacht | outSingapore_109 | 187,520 | 385 | 476.4 | 28.0 | 0.81 |
| Singapore Yacht | outSingapore_110 | 98,945 | 337 | 397.6 | 18.9 | 0.85 |
| **Singapore Yacht ALL** | | **109,473** | **232.05** | **280.1** | | **0.81** |
| Singapore Woodlands | out26_07 | 73,399 | 155 | 191.3 | 17.8 | 0.81 |
| Singapore Woodlands | out26_08 | 71,337 | 138 | 163.0 | 13.0 | 0.85 |
| Singapore Woodlands | out26_09 | 52,613 | 128 | 134.7 | 4.6 | 0.95 |
| Singapore Woodlands | out26_10 | 49,732 | 128 | 149.2 | 11.5 | 0.86 |
| Singapore Woodlands | out26_11 | 87,951 | 159 | 181.9 | 11.0 | 0.87 |
| Singapore Woodlands | out26_12 | 89,753 | 154 | 170.3 | 8.4 | 0.90 |
| Singapore Woodlands | out26_13 | 111,284 | 158 | 192.4 | 17.5 | 0.82 |
| Singapore Woodlands | out26_14 | 55,814 | 133 | 138.1 | 3.8 | 0.96 |
| Singapore Woodlands | out35_15 | 89,158 | 135 | 156.2 | 11.5 | 0.86 |
| Singapore Woodlands | out35_16 | 36,031 | 119 | 146.0 | 13.8 | 0.82 |
| Singapore Woodlands | out35_17 | 11,140 | 79 | 106.1 | 14.2 | 0.74 |
| Singapore Woodlands | out35_18 | 17,807 | 96 | 132.3 | 17.8 | 0.73 |
| Singapore Woodlands | out35_19 | 23,000 | 78 | 94.2 | 9.3 | 0.83 |
| Singapore Woodlands | out35_20 | 8,965 | 77 | 112.1 | 18.3 | 0.69 |
| Singapore Woodlands | out35_21 | 4,840 | 51 | 70.4 | 12.6 | 0.72 |
| Singapore Woodlands | out35_22 | 26,491 | 92 | 121.3 | 15.0 | 0.76 |
| Singapore Woodlands | out35_23 | 120,660 | 150 | 177.0 | 13.5 | 0.85 |
| Singapore Woodlands | out35_24 | 36,294 | 104 | 131.6 | 15.1 | 0.79 |
| Singapore Woodlands | out35_25 | 10,494 | 80 | 109.5 | 15.5 | 0.73 |
| Singapore Woodlands | out35_26 | 18,195 | 87 | 100.6 | 8.0 | 0.87 |
| **Singapore Woodlands ALL** | | **49,748** | **115.1** | **138.9** | | **0.82** |
| Adelaide Container Channel | out45_11 | 217,492 | 387 | 461.5 | 20.5 | 0.84 |
| Adelaide Container Channel | out45_12 | 203,253 | 284 | 371.0 | 24.6 | 0.77 |
| Adelaide Container Channel | out45_13 | 188,260 | 337 | 501.4 | 41.4 | 0.67 |
| Adelaide Container Channel | out45_14 | 102,667 | 266 | 367.1 | 29.1 | 0.72 |
| Adelaide Container Channel | out45_15 | 222,905 | 339 | 485.3 | 36.2 | 0.70 |
| Adelaide Container Channel | out45_16 | 76,976 | 316 | 433.9 | 31.7 | 0.73 |
| Adelaide Container Channel | out45_17 | 120,348 | 306 | 349.6 | 13.3 | 0.88 |
| Adelaide Container Channel | out45_18 | 129,800 | 243 | 290.3 | 15.4 | 0.84 |
| Adelaide Container Channel | out45_19 | 123,280 | 292 | 357.3 | 18.5 | 0.82 |
| **Adelaide Container Channel ALL** | | **153,887** | **307.8** | **401.9** | | **0.77** |
| Adelaide Container Dock 1 | out45_30 | 233,878 | 387 | 502.0 | 28.6 | 0.77 |
| Adelaide Container Dock 1 | out45_31 | 141,913 | 255 | 322.3 | 20.9 | 0.79 |
| Adelaide Container Dock 1 | out45_32 | 157,419 | 264 | 389.8 | 34.7 | 0.68 |
| Adelaide Container Dock 1 | out45_33 | 122,237 | 163 | 192.1 | 11.7 | 0.85 |
| Adelaide Container Dock 1 | out45_34 | 110,838 | 295 | 364.9 | 19.4 | 0.81 |
| Adelaide Container Dock 1 | out45_35 | 114,987 | 250 | 367.5 | 32.5 | 0.68 |
| Adelaide Container Dock 1 | out45_36 | 56,519 | 195 | 249.3 | 18.0 | 0.78 |
| Adelaide Container Dock 1 | out45_37 | 168,697 | 270 | 353.1 | 23.6 | 0.76 |
| Adelaide Container Dock 1 | out45_38 | 122,220 | 280 | 408.8 | 34.5 | 0.68 |
| **Adelaide Container Dock 1 ALL** | | **136,523** | **262.1** | **350.0** | | **0.76** |
| Adelaide Container Dock 2 | out45_29 | 114,297 | 242 | 325.4 | 25.2 | 0.74 |
| Adelaide Container Dock 2 | out45_39 | 133,159 | 319 | 395.1 | 20.8 | 0.81 |

| Site | Sample ID | Number of Reads | Observed metMOTUs | Chao1 Estimate | Chao 1 Std. Error | Observed/Chao1 |
|---|---|---|---|---|---|---|
| Adelaide Container Dock 2 | out45_40 | 142,557 | 309 | 401.0 | 27.0 | 0.77 |
| Adelaide Container Dock 2 | out45_41 | 105,821 | 268 | 358.3 | 27.0 | 0.75 |
| Adelaide Container Dock 2 | out45_42 | 96,446 | 255 | 395.6 | 42.4 | 0.64 |
| Adelaide Container Dock 2 | out45_43 | 101,400 | 230 | 310.2 | 25.5 | 0.74 |
| Adelaide Container Dock 2 | out45_44 | 118,368 | 273 | 348.5 | 22.0 | 0.78 |
| Adelaide Container Dock 2 | out45_45 | 126,677 | 297 | 370.1 | 23.0 | 0.80 |
| Adelaide Container Dock 2 | out45_46 | 110,466 | 293 | 368.5 | 22.0 | 0.80 |
| Adelaide Container Dock 2 | out45_47 | 115,019 | 290 | 406.9 | 32.2 | 0.71 |
| **Adelaide Container Dock 2 ALL** | | **116,421** | **277.6** | **367.9** | | **0.75** |
| Adelaide Fuel Channel | out44_10 | 254,067 | 335 | 436.3 | 27.5 | 0.77 |
| Adelaide Fuel Channel | out44_11 | 230,811 | 273 | 390.0 | 33.2 | 0.70 |
| Adelaide Fuel Channel | out44_12 | 151,807 | 221 | 292.1 | 22.7 | 0.76 |
| Adelaide Fuel Channel | out44_13 | 192,567 | 267 | 336.8 | 20.8 | 0.79 |
| Adelaide Fuel Channel | out44_14 | 127,248 | 274 | 387.4 | 34.0 | 0.71 |
| Adelaide Fuel Channel | out44_16 | 149,931 | 287 | 395.0 | 29.1 | 0.73 |
| Adelaide Fuel Channel | out44_17 | 142,095 | 306 | 391.0 | 24.1 | 0.78 |
| Adelaide Fuel Channel | out44_18 | 205,329 | 329 | 400.2 | 19.8 | 0.82 |
| Adelaide Fuel Channel | out44_19 | 291,024 | 352 | 421.0 | 19.5 | 0.84 |
| **Adelaide Fuel Channel ALL** | | **193,875** | **293.8** | **383.3** | | **0.77** |
| Adelaide Fuel Dock | out44_1 | 100,172 | 270 | 327.5 | 17.5 | 0.82 |
| Adelaide Fuel Dock | out44_2 | 111,387 | 334 | 427.1 | 25.0 | 0.78 |
| Adelaide Fuel Dock | out44_3 | 170,038 | 332 | 387.2 | 16.5 | 0.86 |
| Adelaide Fuel Dock | out44_4 | 218,584 | 290 | 397.8 | 30.4 | 0.73 |
| Adelaide Fuel Dock | out44_5 | 276,160 | 400 | 462.4 | 17.5 | 0.87 |
| Adelaide Fuel Dock | out44_6 | 171,561 | 366 | 460.0 | 24.3 | 0.80 |
| Adelaide Fuel Dock | out44_7 | 217,419 | 389 | 460.3 | 19.4 | 0.85 |
| Adelaide Fuel Dock | out44_8 | 141,807 | 288 | 337.2 | 15.2 | 0.85 |
| Adelaide Fuel Dock | out44_9 | 183,457 | 315 | 402.8 | 23.5 | 0.78 |
| Adelaide Fuel Dock | out45_28 | 125,696 | 286 | 357.9 | 20.3 | 0.80 |
| **Adelaide Fuel Dock ALL** | | **171,628** | **327.0** | **402.0** | | **0.81** |
| Adelaide Marina Channel | out45_1 | 150,588 | 361 | 483.6 | 32.7 | 0.75 |
| Adelaide Marina Channel | out45_2 | 153,276 | 305 | 366.3 | 18.2 | 0.83 |
| Adelaide Marina Channel | out45_27 | 136,365 | 281 | 350.3 | 21.3 | 0.80 |
| Adelaide Marina Channel | out45_3 | 135,541 | 377 | 464.3 | 23.9 | 0.81 |
| Adelaide Marina Channel | out45_4 | 182,217 | 387 | 483.1 | 24.5 | 0.80 |
| Adelaide Marina Channel | out45_5 | 212,373 | 344 | 391.5 | 14.3 | 0.88 |
| Adelaide Marina Channel | out45_6 | 159,465 | 335 | 396.6 | 17.5 | 0.84 |
| Adelaide Marina Channel | out45_7 | 194,245 | 352 | 404.4 | 15.9 | 0.87 |
| Adelaide Marina Channel | out45_8 | 136,813 | 339 | 460.5 | 30.3 | 0.74 |
| Adelaide Marina Channel | out45_9 | 199,106 | 328 | 431.6 | 28.2 | 0.76 |
| **Adelaide Marina Channel ALL** | | **165,999** | **340.9** | **423.2** | | **0.81** |
| Adelaide Marina Dock | out44_39 | 107,569 | 227 | 256.7 | 11.3 | 0.88 |
| Adelaide Marina Dock | out44_40 | 40,591 | 152 | 197.2 | 17.1 | 0.77 |
| Adelaide Marina Dock | out44_41 | 169,749 | 266 | 333.3 | 20.9 | 0.80 |
| Adelaide Marina Dock | out44_42 | 190,478 | 352 | 386.5 | 12.7 | 0.91 |
| Adelaide Marina Dock | out44_43 | 103,057 | 296 | 332.9 | 12.5 | 0.89 |
| Adelaide Marina Dock | out44_45 | 419,323 | 393 | 433.7 | 13.9 | 0.91 |
| Adelaide Marina Dock | out44_46 | 226,087 | 340 | 382.4 | 14.6 | 0.89 |
| Adelaide Marina Dock | out44_47 | 281,467 | 359 | 454.2 | 26.5 | 0.79 |
| Adelaide Marina Dock | out44_48 | 158,612 | 271 | 350.1 | 23.0 | 0.77 |
| **Adelaide Marina Dock ALL** | | **188,548** | **295.1** | **347.4** | | **0.85** |

**Supplementary Table S4.** Known non-indigenous species (NIS) detection from ports Chicago and Adelaide. Accession (Genbank accession number), % Coverage, % Identity and e-value refer to the Geneious assignment statistics for the given NIS detection. The number of reads per NIS MOTU and the reference for the non-indigenous species status in a given port are also noted. References are (1) NOAA Great Lakes Nonindigenous Species Information System at https://www.glerl.noaa.gov/glansis/, (2) Vasquez, Adrian A., *et al.* "*Eurytemora carolleeae* in the Laurentian Great Lakes revealed by phylogenetic and morphological analysis." *Journal of Great Lakes research* 42.4 (2016): 802. (3) Invasive Species Compendium at www.cabi.org, and (4) Wiltshire, K., K. Rowling, and M. Deveney. (2010). Introduced marine species in South Australia: a review of records and distribution mapping. South Australia Research and Development Institute (Aquatic Sciences), Adelaide,. SARDI Publication No. F2010/000305-1. SARDI Research Report Series No. 468. 232p

| **Port** | **Bar-code** | **OTU ID** | **Species** | **Accession** | **% Coverage** | **% Ident.** | **e-value** | **# Reads** | **Notes (Reference)** |
|---|---|---|---|---|---|---|---|---|---|
| Chicago | 18S | 7975 | *Dreissena rostriformis* | JX099479 | 1 | 0.889 | 1.5E-117 | 2 | Quagga Mussel (1) |
| Chicago | 18S | 31 | *Eurytemora affinis* | JX995299 | 1 | 1 | 0 | 174,840 | copepod (1) |
| Chicago | COI | 166 | *Corbicula fluminea* | KU318325 | 1 | 1 | 1.63E-161 | 1 | Asian Clam (1) |
| Chicago | COI | 6316 | *Cyprinus carpio* | KU146530 | 1 | 1 | 1.63E-161 | 8 | Common Carp (1) |
| Chicago | COI | 24 | *Eurytemora carolleeae* | KR611038 | 1 | 0.997 | 7.58E-169 | 124,156 | copepod (2) |
| Chicago | COI | 3262 | *Lumbricus rubellus* | KM612229 | 1 | 1 | 1.65E-161 | 49 | European earthworm (3) |
| Chicago | COI | 11177 | *Morone americana* | KU641485 | 1 | 1 | 1.65E-161 | 5 | White Perch (1) |
| Adelaide | 18S | 2408 | *Ciona intestinalis* | AK173369 | 1 | 1 | 8.18E-180 | 1 | (4) |
| Adelaide | 18S | 3495 | *Coryne eximia* | GQ424325 | 1 | 0.997 | 1.78E-176 | 1 | (4) |
| Adelaide | 18S | 90 | *Hydroides elegans* | KP178705 | 1 | 0.997 | 2.28E-180 | 1 | (4) |
| Adelaide | 18S | 1130 | *Hydroides elegans* | KP178705 | 0.9457 | 0.997 | 7.77E-170 | 0.9457 | (4) |
| Adelaide | 18S | 1618 | *Hydroides elegans* | KP178705 | 0.9429 | 0.991 | 6.05E-166 | 0.9429 | (4) |
| Adelaide | 18S | 3831 | *Hydroides elegans* | KP178705 | 0.9286 | 0.997 | 1.80E-166 | 0.9286 | (4) |
| Adelaide | 18S | 4946 | *Hydroides elegans* | KP178705 | 1 | 0.892 | 1.09E-118 | 1 | (4) |
| Adelaide | 18S | 6270 | *Hydroides elegans* | KP178705 | 1 | 0.943 | 1.13E-143 | 1 | (4) |
| Adelaide | 18S | 519 | *Musculista senhousia* | AB201231 | 1 | 1 | 8.21E-180 | 1 | (4) |
| Adelaide | 18S | 1488 | *Plumularia setacea* | KT722424 | 0.9972 | 0.997 | 0 | 0.9972 | (4) |
| Adelaide | 18S | 1928 | *Styela plicata* | KJ720706 | 1 | 0.994 | 1.66E-176 | 1 | (4) |
| Adelaide | COI | 11696 | *Carcnus maenas* | KT952478 | 1 | 1 | 1.63E-161 | 1 | (4) |
| Adelaide | COI | 2928 | *Cordylophora caspia* | KU695595 | 0.9489 | 0.775 | 3.27E-39 | 0.9489 | (4) |
| Adelaide | COI | 10261 | *Cordylophora caspia* | KU695595 | 0.9585 | 0.756 | 7.12E-31 | 0.9585 | (4) |
| Adelaide | COI | 4711 | *Coryne eximia* | KT981902 | 0.9457 | 0.828 | 5.18E-67 | 0.9457 | (4) |
| Adelaide | COI | 5625 | *Coryne eximia* | KT981909 | 0.9361 | 0.83 | 5.18E-67 | 0.9361 | (4) |
| Adelaide | COI | 10467 | *Coryne eximia* | KT981902 | 0.8626 | 0.833 | 1.47E-62 | 0.8626 | (4) |
| Adelaide | COI | 2806 | *Hydroides elegans* | JQ885939 | 1 | 0.997 | 7.66E-160 | 1 | (4) |
| Adelaide | COI | 6493 | *Hydroides elegans* | JQ885939 | 0.9968 | 0.894 | 6.33E-106 | 0.9968 | (4) |
| Adelaide | COI | 2348 | *Musculista senhousia* | HG005372 | 0.8115 | 0.797 | 5.43E-42 | 0.8115 | (4) |
| Adelaide | COI | 40 | *Mytilus galloprovincialis* | GQ468291 | 1 | 1 | 1.63E-161 | 1 | (4) |
| Adelaide | COI | 2011 | *Mytilus galloprovincialis* | FJ890850 | 1 | 0.971 | 5.98E-146 | 1 | (4) |
| Adelaide | COI | 903 | *Mytilus galloprovincialis* | FJ890850 | 0.9649 | 0.99 | 2.14E-150 | 0.9649 | (4) |

| Port | Bar-code | OTU ID | Species | Accession | % Coverage | % Ident. | e-value | # Reads | Notes (Reference) |
|---|---|---|---|---|---|---|---|---|---|
| Adelaide | COI | 524 | *Mytilus galloprovincialis* | AY363687 | 0.9808 | 0.98 | 3.58E-148 | 0.9808 | (4) |
| Adelaide | COI | 5175 | *Mytilus galloprovincialis* | HM140776 | 1 | 0.971 | 1.66E-146 | 1 | (4) |
| Adelaide | COI | 2759 | *Mytilus galloprovincialis* | KT988324 | 1 | 1 | 1.65E-161 | 1 | (4) |
| Adelaide | COI | 6655 | *Mytilus galloprovincialis* | AY363687 | 1 | 0.955 | 3.63E-138 | 1 | (4) |
| Adelaide | COI | 5061 | *Mytilus galloprovincialis* | HM140776 | 0.777 | 0.948 | 1.93E-41 | 0.777 | (4) |
| Adelaide | COI | 6626 | *Mytilus galloprovincialis* | HM140776 | 0.9393 | 0.952 | 1.32E-127 | 0.9393 | (4) |
| Adelaide | COI | 4320 | *Plumularia setacea* | KF982163 | 1 | 0.917 | 3.73E-118 | 1 | (4) |
| Adelaide | COI | 10534 | *Plumularia setacea* | KF982171 | 0.9457 | 0.815 | 2.45E-60 | 0.9457 | (4) |
| Adelaide | COI | 2094 | *Styela plicata* | KT988335 | 1 | 1 | 1.63E-161 | 1 | (4) |
| Adelaide | COI | 3614 | *Tricellaria occidentalis* | HQ896152 | 0.5655 | 0.96 | 3.07E-74 | 0.5655 | (4) |
| Adelaide | COI | 5616 | *Tridentiger trigonocephalus* | KT282115 | 0.9968 | 0.949 | 2.82E-134 | 0.9968 | (4) |
| Adelaide | COI | 7486 | *Watersipora arcuata* | AF441089 | 1 | 0.994 | 3.53E-158 | 1 | (4) |

**Supplementary Table S5.** Metazoan MOTUs found in three or more ports. Accession (Genbank accession number), % Coverage, % Identity and e-value refer to the Geneious assignment statistics for the given OTU. The number of port (# ports) in which at least one read of each MOTU was found and the total read counts for each port are given. References: (1) FishBase at www.fishbase.org, (2) World Mollusc Species Database at http://www.bagniliggia.it/WMSD/Lindex_aaa.htm, (3) World Register of Marine Species at www.marinespecies.org, (4) Global Biodiversity Information Facility at www.gbif.org, (5) NCBI Genbank at https://www.ncbi.nlm.nih.gov/genbank/, (6) Integrated Taxonomic Information System at www.itis.gov, (7) World Spider Catalog at https://wsc.nmbe.ch, and (8) the IUCN Red List at www.iucnredlist.org.

| Bar-code | OTU ID | Species | Accession | % Coverage | % Identity | # Ports | Chicago Reads | Churchill Reads | Adelaide Reads | Singapore Reads | Notes (Reference) |
|---|---|---|---|---|---|---|---|---|---|---|---|
| 18S | 64 | *Abudefduf septemfasciatus* | KX015768 | 1 | 1 | 3 | 0 | 227 | 7 | 218 | Banded Sergeant (fish) from Indo-Pacific (1) |
| 18S | 266 | *Acanthochitona rhodea* | HQ907736 | 1 | 1 | 3 | 0 | 5 | 13 | 6064 | marine chiton known from west Africa (2) |
| 18S | 527 | *Aurelia limbata* | JX393277 | 0.9884 | 0.997 | 3 | 0 | 4511 | 12 | 1 | marine scyphozoan from Arctic and north Atlantic (3) |
| 18S | 1869 | *Bougainvillia muscus* | KT722388 | 1 | 0.985 | 4 | 1 | 14 | 133 | 15 | cosmopolitan marine hydroid (3) |
| 18S | 1745 | *Cephalodella forficula* | DQ297693 | 0.9914 | 0.974 | 4 | 1 | 1203 | 2 | 650 | cosmopolitan rotifer (4) |
| 18S | 244 | *Critomolgus sp. 2 New Caledonia-RJH-2004* | AY627009 | 1 | 0.951 | 3 | 2 | 0 | 5 | 4296 | marine copepod from New Caledonia (5) |
| 18S | 1427 | *Dero furcata* | HQ691214 | 1 | 0.991 | 3 | 27 | 210 | 0 | 3 | freshwater oligochaete from North American (7) |
| 18S | 1013 | *Encentrum astridae* | DQ297695 | 1 | 0.98 | 4 | 1351 | 2 | 6 | 33 | marine rotifer, little information online (5) |
| 18S | 948 | *Euchlanis dilatata* | AY218116 | 0.9885 | 0.983 | 3 | 631 | 0 | 2 | 2 | freshwater rotifer from Europe (3) |
| 18S | 108 | *Eudendrium carneum* | KT722396 | 1 | 1 | 3 | 1 | 372 | 0 | 24284 | marine hyroid from Europe, Australia, possibly North America (3) |
| 18S | 2637 | *Euphysa aurata* | EU876562 | 1 | 1 | 3 | 0 | 12 | 23 | 5 | marine hydroid from Artic and Atlantic (3) |
| 18S | 721 | *Forcepia sp. 0CDN7230-S* | KC902407 | 1 | 1 | 3 | 0 | 1 | 317 | 401 | marine sponge from South Africa (5) |

| Bar-code | OTU ID | Species | Accession | % Coverage | % Identity | # Ports | Chicago Reads | Churchill Reads | Adelaide Reads | Singapore Reads | Notes (Reference) |
|---|---|---|---|---|---|---|---|---|---|---|---|
| 18S | 3121 | *Glycera americana* | KT989351 | 1 | 1 | 3 | 0 | 5 | 11 | 8 | cosmopolitan marine polychaete (3) |
| 18S | 4759 | *Homo sapiens* | AC097532 | 1 | 1 | 4 | 1 | 1 | 1 | 6 | human (5) |
| 18S | 199 | *Hymeniacidon perlevis* | KC902358 | 1 | 1 | 3 | 0 | 26 | 1647 | 6160 | cosmopolitan marine sponge (3) |
| 18S | 3618 | *Keratella quadrata* | KX358062 | 1 | 0.968 | 3 | 372 | 2 | 0 | 15 | freshwater rotifer from Europe (3) |
| 18S | 6366 | *Leucilla sp. OV-2012* | JQ272323 | 0.9913 | 0.982 | 3 | 0 | 1 | 9 | 47 | marine sponge accession from Caribbean (5) |
| 18S | 372 | *Leucosolenia sp. QN-2004-1* | AJ622898 | 1 | 1 | 3 | 0 | 1 | 1419 | 142 | marine sponge accession from MA, USA (5) |
| 18S | 1848 | *Macrostomum rubrocinctum* | KC869789 | 1 | 1 | 3 | 3 | 3 | 0 | 73 | marine flatworm from Canada & Europe '(3) |
| 18S | 2026 | *Microstomum lineare* | KP730484 | 1 | 1 | 4 | 14 | 53 | 6 | 5 | cosmopolitan flatworm; freshwater, estuarine, & marine habitats (3) |
| 18S | 462 | *Monostyla sp. MPN-2013* | KF159017 | 1 | 0.997 | 4 | 1678 | 1 | 14 | 18 | rotifer sequenced at Hamburg museum (5) |
| 18S | 5166 | *Notommata cordonella* | DQ297711 | 0.9425 | 0.985 | 4 | 364 | 2 | 314 | 41 | rotifer, little information online (5) |
| 18S | 120 | *Notommata cordonella* | DQ297711 | 0.9454 | 0.976 | 3 | 139844 | 2104 | 13 | 0 | rotifer, little information online (5) |
| 18S | 1674 | *Notommata cordonella* | DQ297711 | 0.9425 | 0.976 | 3 | 190 | 24 | 16 | 0 | rotifer, little information online (5) |
| 18S | 7196 | *Notommata cordonella* | DQ297711 | 1 | 0.946 | 3 | 20 | 1 | 1 | 0 | rotifer, little information online (5) |
| 18S | 337 | *Paraleucilla sp. MM-2002* | AF452023 | 1 | 0.997 | 3 | 0 | 8 | 3562 | 800 | calcareous sponge from France (5) |
| 18S | 525 | *Polyarthra remata* | DQ297716 | 1 | 0.966 | 3 | 15568 | 2 | 0 | 4 | freshwater rotifer from Europe (3) |
| 18S | 331 | *Scissula similis* | KC429394 | 1 | 1 | 3 | 0 | 230 | 591 | 2865 | marine bivalve from Gulf of Mexico (3) |
| 18S | 1773 | *Solmundella bitentaculata* | EU247812 | 1 | 1 | 3 | 0 | 113 | 2 | 9 | cosmopolitan marine hydroid (3) |

| Bar-code | OTU ID | Species | Accession | % Coverage | % Identity | # Ports | Chicago Reads | Churchill Reads | Adelaide Reads | Singapore Reads | Notes (Reference) |
|---|---|---|---|---|---|---|---|---|---|---|---|
| 18S | 3074 | *Spongionella cf. foliascens 0CDN9587-G* | KC902176 | 0.6958 | 0.852 | 3 | 14 | 3 | 0 | 10 | marine sponge accession from Palau (5) |
| 18S | 363 | *Styela gibbsii* | FM897319 | 1 | 0.977 | 3 | 0 | 1 | 197 | 3037 | ascidian from Northeast Pacific (3) |
| 18S | 19 | *Synchaeta pectinata* | KP875584 | 1 | 1 | 4 | 198763 | 1594 | 989 | 613 | cosmopolitan rotifer found in freshwater, estuarine, and marine (3) |
| 18S | 2454 | *Synchaeta pectinata* | KF561106 | 1 | 0.974 | 3 | 2 | 51 | 1 | 0 | cosmopolitan rotifer; freshwater, estuarine, and marine (3) |
| COI | 2843 | *Alticini sp. 8 BT-2014* | KJ677711 | 0.8594 | 0.777 | 3 | 43 | 1 | 1 | 0 | flea beetle accession from Ecuador (5) |
| COI | 504 | *Antocha sp. SS-2012* | KC263146 | 0.8722 | 0.791 | 3 | 0 | 258 | 466 | 468 | crane fly (5) |
| COI | 5042 | *Aulonia albimana* | KX537209 | 0.5144 | 0.876 | 3 | 1 | 0 | 4 | 15 | Palearctic spider (7) |
| COI | 1515 | *Bulinus sp. BfSite13* | AM921819 | 0.901 | 0.816 | 3 | 0 | 50 | 42 | 42 | tropical freshwater snail from Uganda (5) |
| COI | 1237 | *Cubaia aphrodite* | JN700942 | 0.7955 | 0.779 | 3 | 26 | 192 | 1 | 0 | marine hydroid from Belize (4) |
| COI | 4292 | *Eucheilota maculata* | KC440070 | 0.9553 | 0.849 | 3 | 0 | 8 | 5 | 12 | European hydroid, marine and freshwater '(3) |
| COI | 901 | *Falculea palliata* | JQ239296 | 0.8786 | 0.738 | 3 | 18 | 3 | 360 | 0 | bird endemic to Madagascar (8) |
| COI | 6542 | *Haliclona oculata* | HQ379430 | 1 | 0.987 | 3 | 0 | 1 | 8 | 1 | cosmopolitan marine sponge '(3) |
| COI | 1023 | *Macrothrix sp. HE-364* | KC617066 | 1 | 0.994 | 3 | 3 | 13 | 0 | 268 | cladoceran, accession from Mexico (5) |
| COI | 613 | *Macrothrix sp. HE-364* | KC617066 | 0.9968 | 0.926 | 3 | 74 | 21 | 0 | 402 | cladoceran accession from Mexico (5) |
| COI | 1007 | *Poresta sp. 'lophocera'* | JN807164 | 0.8978 | 0.794 | 3 | 0 | 62 | 202 | 2 | moth from Costa Rica (5) |
| COI | 6439 | *Sernokorba pallidipatellis* | JN817229 | 0.8786 | 0.778 | 3 | 12 | 1 | 0 | 2 | spider from Asia (7) |
| COI | 1682 | *Sus scrofa* | KU556691 | 1 | 1 | 3 | 0 | 2 | 68 | 46 | feral pig, wide distribution (8) |
